# Action-mode subnetworks for decision-making, action control, and feedback

**DOI:** 10.1073/pnas.2502021122

**Published:** 2025-06-30

**Authors:** Carolina Badke D’Andrea, Timothy O. Laumann, Dillan J. Newbold, Charles J. Lynch, Mohammad Hadji, Steven M. Nelson, Ashley N. Nielsen, Roselyne J. Chauvin, Samuel R. Krimmel, Abraham Z. Snyder, Scott Marek, Deanna J. Greene, Marcus E. Raichle, Nico U. F. Dosenbach, Evan M. Gordon

**Affiliations:** ^a^Department of Radiology, Washington University School of Medicine, St. Louis, MO 63110; ^b^Department of Psychiatry, Washington University School of Medicine, St. Louis, MO 63110; ^c^Department of Cognitive Science, University of California San Diego, La Jolla, CA 92093; ^d^Department of Neurology, New York University Medical Center, New York, NY 10016; ^e^Department of Psychiatry, Weill Cornell Medicine, New York, NY 10065; ^f^Department of Pediatrics, University of Minnesota Medical School, Minneapolis, MN 55455; ^g^Department of Neurology, Washington University School of Medicine, St. Louis, MO 63110; ^h^Artificial Intelligence for Health Institute, Washington University School of Medicine, St. Louis, MO 63110; ^i^Department of Biomedical Engineering, Washington University in St. Louis, St. Louis, MO 63130; ^j^Department of Psychological and Brain Sciences, Washington University School of Medicine, St. Louis, MO 63110; ^k^Department of Neuroscience, Washington University School of Medicine, St. Louis, MO 63110; ^l^Program in Occupational Therapy, Washington University School of Medicine, St. Louis, MO 63110; ^m^Department of Pediatrics, Washington University School of Medicine, St. Louis, MO 63110

**Keywords:** action-mode network, cognitive control, functional connectivity, action control, precision functional mapping

## Abstract

The action-mode network (AMN) is a canonical functional brain network first identified using resting-state functional connectivity (RSFC). Based on animal and human data, we have proposed that AMN supports the brain’s action mode by controlling functions required for successful goal-directed behavior. However, task fMRI averaged across groups has associated AMN regions with a variety of behaviors, contributing to uncertainty about AMN function. Here, we investigated the AMN using an inside-out approach, in which the network architecture of the AMN is first precisely mapped within individuals and then associated with behavioral functions. Individual-specific precision functional mapping with >5 h of RSFC and task functional magnetic resonance imaging (fMRI) data revealed a replicable AMN subnetwork structure. AMN subnetworks were characterized and annotated by combining a meta-analytic network association method with RSFC, intrinsic timing, and task activation profiling. We demonstrate the existence of AMN-Decision, -Action, and -Feedback subnetworks that are distributed across lobes, forming a temporally sequential within-network processing stream by which the brain adjudicates between possible goals, sets action plans, and modifies those plans in response to feedback such as pain. A subnetwork in the pars marginalis of the cingulate was distinct from the Decision, Action, and Feedback subnetworks and may be important for the construction of the bodily self.

## Significance

The human brain is organized into large networks. One important brain network is the Action Mode Network, which controls functions related to goal-directed behavior. In group-averaged data, this network emerges as a unitary whole, despite its involvement in a variety of behaviors. Here, we tested whether Action Mode networks found in individual humans, rather than group-average networks, might contain organized substructure that helps explain its functional heterogeneity. In individuals, we identified four subnetworks within the Action Mode Network related to decisions, action implementation, feedback, and the bodily self. Together, these subnetworks form a processing stream by which the brain decides goals, sets action plans, and modifies those plans in response to feedback such as pain.

The human brain is organized into about a dozen canonical functional networks that have been repeatedly verified across populations, datasets, and analyses (1–7). One of the first functional networks discovered was the action-mode network (AMN) (8), which was originally referred to as the cingulo-opercular network (9–11) based on its neuroanatomy. AMN is composed of functionally coupled regions in the dorsal anterior cingulate cortex (dACC), supplementary motor area (SMA), anterior and middle insula, supramarginal gyrus (SMG), pars marginalis of the cingulate gyrus, anterior prefrontal cortex (aPFC), anterior putamen, the central portion of the thalamus (in or near the ventral intermediate nucleus), and lateral cerebellum (5, 6, 9–13). The AMN has often been confused with the Salience network (14), though these two networks are anatomically and functionally distinct. Relative to AMN, Salience is localized more anteriorly in dACC and inferiorly in anterior insula and exhibits subcortical connectivity [to nucleus accumbens rather than anterior putamen (15, 16)] and function [reward and motivation (17, 18)] inconsistent with the AMN. Animal and human studies suggest that the AMN initiates and maintains the brain’s action mode, a state in which arousal is heightened, attention is externally focused, and action plans are created and converted to movements (8, 9, 19–23). Lesions to AMN regions impoverish voluntary behavior while preserving other functions (24, 25). Thus, AMN appears to control and enable behavior expressed as physical actions—the most important outcome of any set of complex brain processes.

Task fMRI research has associated AMN regions with a wide variety of behaviors, leading to significant uncertainly about AMN function. The AMN coactivates (along with several other brain networks) across a wide variety of cognitive tasks, leading to high-level characterizations of task-positive activity as reflecting a broad extrinsic-mode network (26, 27). The more restricted set of AMN regions was initially characterized as a network critical for exerting top–down control over other cognitive functions. Large signals are observed in the AMN when complex cognitive tasks are initiated (9, 28, 29), and it exhibits sustained signals related to task goal maintenance (9–11). AMN regions also respond strongly to feedback, enabling more effective control in the future. The AMN exhibits large error responses; its activity is modulated by trial difficulty (i.e., time on task); and AMN regions are more strongly activated by ambiguous stimuli and response conflict (9–11, 30–40). These data argue for the AMN as an executive control network.

However, other lines of work suggest that some AMN regions are involved in motor control (41, 42). DACC and mid-insula have been linked to urges for motor action (43, 44). Dorsomedial prefrontal AMN regions (dACC, pre-SMA, SMA) exhibit strong functional connectivity with the somato-cognitive action network (SCAN), which alternates with effector-specific regions in the primary motor cortex (M1) (45). These SCAN–AMN connections are thought to represent a link through which whole-body action plans are realized as movements (45). Prolonged arm immobilization results in both substantial changes in motor behavior and strengthened functional connectivity between disused M1 and the AMN, suggesting an important role in motor control (46, 47). In nonhuman primates, the rostral, ventral, and dorsal cingulate motor areas are critical for motor planning, as movement-preceding signals appear in these cingulate areas prior to SMA and M1 (48, 49); their human homologs are likely located within the dACC portion of the AMN.

The AMN also plays a role in processing painful stimuli. The dACC, insula, and SMG/inferior parietal lobule are commonly reported as brain regions most active during application of painful stimuli (50–55). This pattern is consistent across both somatic and visceral pain (56) and across both healthy and chronic pain patients (55). This pattern is spatially distinct from representations of negative affect or social pain (52) and partially overlaps with AMN regions involved in cognitive control (57).

Traditional task-based approaches for understanding the function of brain regions follow an “outside-in” framework, in which discrete behavioral functions are derived a priori, via introspection and psychology, and each function is separately investigated and localized in the brain. Each separate outside-in study typically succeeds in identifying brain regions and networks associated with the function of interest. However, reconciling findings across studies is difficult when multiple a priori functions map to the same brain region. If the dACC region exhibits signals related to executive control (9–11, 39), task initiation (9, 28, 29), arousal (21, 22), motor control (42, 47, 48), cognitive conflict (36, 40), errors (33), and pain monitoring (52–54), how can a concise account of dACC, and of the broader AMN in which it participates, account for this dizzying range of functionality?

It is instructive to realize that the brain may not necessarily be organized in a fashion convergent with our a priori categories of behavior, but instead follows its own organization, which was developed haphazardly but with purpose by evolutionary pressures (58). Because we do not and cannot know this organization a priori, here we employ an alternative, inside-out approach, as articulated by Buzsaki (59), that first identifies fundamental properties and organizational principles of the brain and then works to understand how they give rise to behaviors (8, 59).

The inside-out framework (59) has produced some of the most significant advances in our understanding of brain organization. For example, the most widely studied brain network, the Default mode network (DMN), was first identified not as regions activated by a specific a priori behavioral function but as regions exhibiting consistent and unexpected deactivation across many tasks (60, 61). Subsequent investigation of DMN using an inside-out approach—assuming the existence of the DMN as a discrete brain network and investigating its functional properties—revealed that the DMN’s functions encompass seemingly disparate behavioral constructs such as episodic memory, planning for the future, processing social interactions, and theory of mind (62–64). With the inside-out approach, the DMN is inferred to be a network broadly subserving self-oriented cognitive function. Other work has taken a similar perspective in lateral prefrontal and parietal cortex, where a domain-general network has been described that activates during many different cognitive functions (27, 39, 65–67). Broadly, in starting with known brain circuits and searching for their associated cognitive functions, the inside-out approach may thus be at least a partial solution to the classic problem of reverse inference in fMRI (68).

The inside-out approach has further revealed that much of the functional heterogeneity of the DMN is enabled by its distinct network substructures. Brain networks such as DMN are not unitary entities, but exist as one level of a nested functional organization (69–73) that can be further divided into distinct substructures. In the DMN, network substructures—subnetworks—are functionally dissociable (73–75), such that they vary in their connectivity to other brain networks (73) and are differentially engaged by various self-oriented cognitive processes (76). They are also temporally dissociable, as subnetwork signals exhibit lagged relationships with each other, suggesting sequentially ordered processing streams (73).

To improve precision of network delineation, our group has continued to advance precision functional mapping (PFM), which uses repeated fMRI scanning to reliably characterize brain networks within individual highly sampled participants (2, 12, 77–87). By characterizing networks at the individual level, detailed subdivisions within large-scale networks have been identified, including within the DMN (73, 74), Dorsal Attention network (DAN) (88), striatum (16), and primary motor cortex (45).

Here, we further advanced our individual-specific PFM approach to characterize and then annotate the AMN’s substructure (>5 h RSFC and >5 h task fMRI per participant; n = 15). We first identified AMN subnetworks from RSFC (16, 73) and then investigated their functions. Meta-analytic tools such as Neurosynth (89) are useful for functional annotation of single brain regions, but not for distributed networks. Thus, we developed a technique termed meta-analytic network association (MANA) to identify Neurosynth functions associated with spatially distributed subnetworks. To further validate functional dissociations between subnetworks, we investigated differences in their whole-brain RSFC patterns; in the lagged temporal ordering of their signals; and in their task activation.

## Results

### Subnetworks within AMN.

**Fig. 1.**
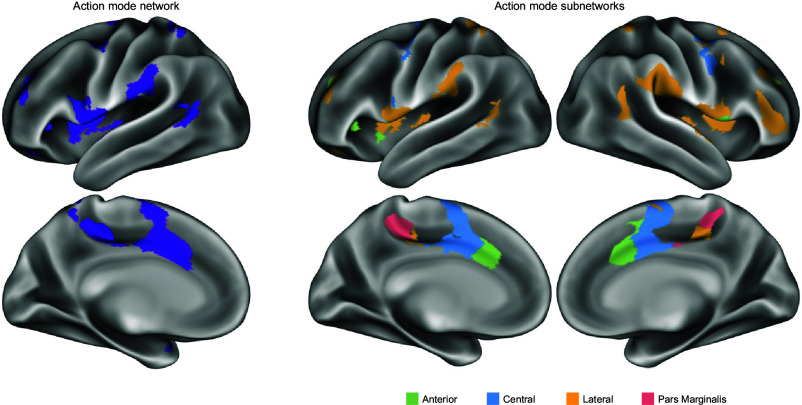
Action-mode subnetworks. The complete AMN (*Left*) and AMN subnetworks (*Right*) in an exemplar participant (P01) with 294 min of resting-state fMRI data. Four distinct subnetworks were identified that matched across individuals: an anterior subnetwork in dorsal anterior cingulate, dorsal anterior insula, and anterior PFC (green); a central subnetwork in more posterior dorsal anterior cingulate extending up to supplementary motor area (blue); a lateral subnetwork in middle insula, supramarginal gyrus, posterior inferior frontal gyrus, and inferior frontal sulcus (yellow); and a subnetwork centered the pars marginalis of the cingulate sulcus (magenta). See *SI Appendix*, Fig. S1 for all individual participants.

The subnetwork structure of each individual’s AMN was identified using an automated detection algorithm. Fine-scale communities were identified at a level previously shown to optimally capture divisions in resting-state and task data relative to a rotation-based null model (73). Communities within AMN were then matched across individuals based on their spatial overlap, and a clustering algorithm sorted the matched communities into distinct subnetworks. Four distinct subnetworks were identified within the AMN (exemplar participant in Fig. 1; all PFM individuals in *SI Appendix*, Fig. S1). The AMN subnetworks differed in their relative prominence in more anterior, central, lateral, and pars marginalis portions of the unified AMN. The anterior subnetwork (green) was most prominent in the dACC, dorsal anterior insula, and aPFC. The central subnetwork (blue) included the posterior dACC and SMA. The lateral subnetwork (blue) was represented in the SMG, middle insula, anterior inferior frontal sulcus, and posterior inferior frontal gyrus. A fourth subnetwork was consistently localized to the pars marginalis of the cingulate sulcus. Three of the four subnetworks (anterior, central, and lateral) were identified in every single individual; in two subjects, the subnetwork in the pars marginalis was incorporated into the lateral subnetwork.

**Fig. 2.**
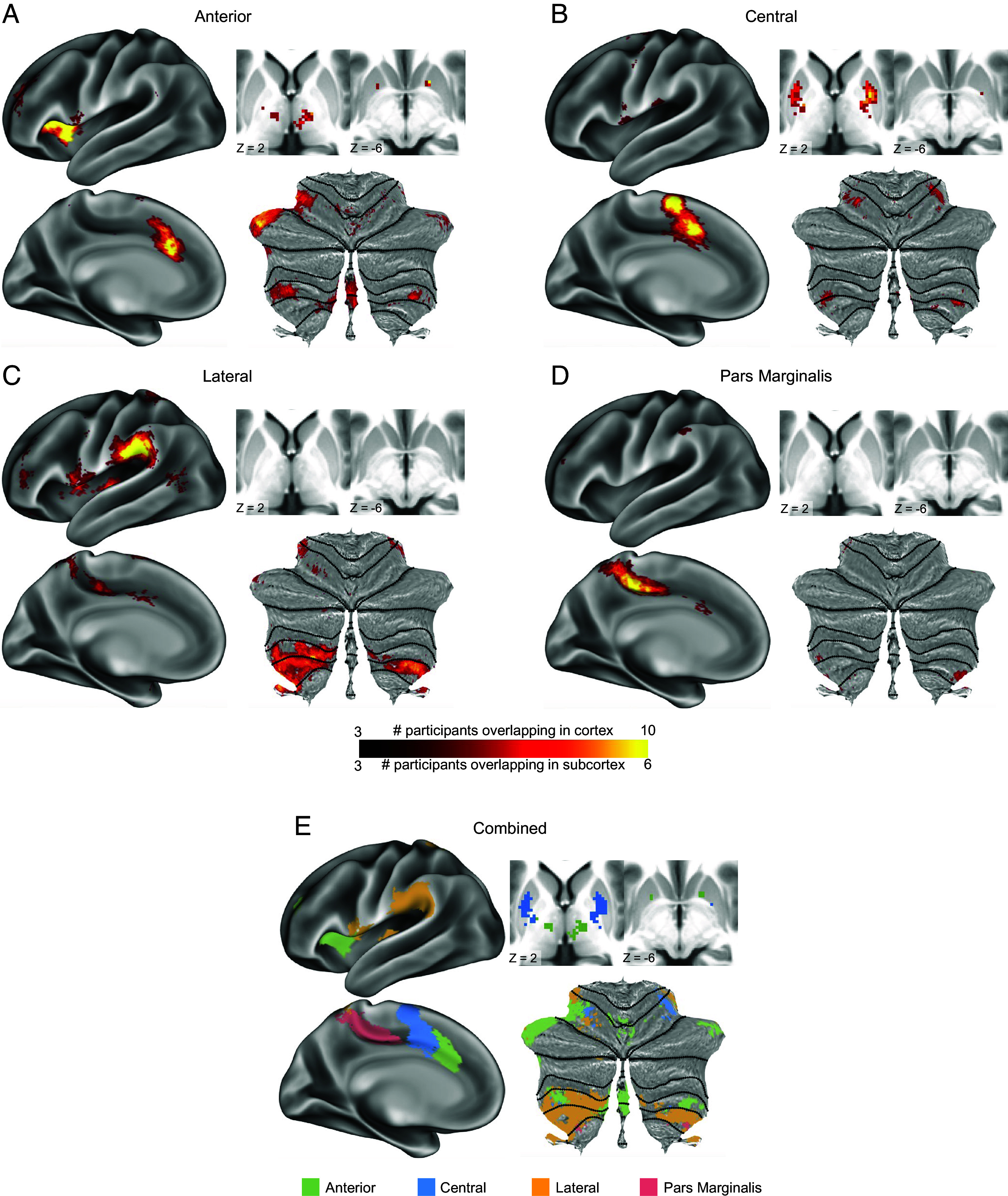
Consistency of cortical and subcortical distribution of AMN subnetworks. (*A*–*D*) Density maps illustrate the number of PFM participants with overlapping subnetwork representations at each point in cortex (*Left*), thalamus and striatum (*Top Right*), and cerebellum (*Bottom Right*; flatmap), for the (*A*) Anterior, (*B*) Central, (*C*) Lateral, and (*D*) Pars Marginalis subnetworks. Maps were thresholded to retain points at which at least three participants exhibited overlap. Differential scaling of density maps in cortex and subcortex was required due to lower signal-to-noise ratio in subcortex increasing the cross-participant variability. (*E*) A winner-take-all map illustrates the relative topographies of all three subnetworks.

The four AMN subnetworks also exhibited significant topological consistency across participants in subcortex (Fig. 2). The anterior subnetwork (Fig. 2*A*) had consistent representation in only a small segment of anterior ventral putamen, adjacent to but not within the nucleus accumbens, but exhibited thalamic representation spanning the ventral lateral anterior and ventral posterior lateral nuclei [localized using the DISTAL atlas (90)]. It was also present in lateral anterior cerebellum and posterior cerebellum. The central subnetwork (Fig. 2*B*) had extensive representation in putamen that was dorsal and posterior of the anterior subnetwork. Relative to the anterior subnetwork, the central subnetwork exhibited more medial representation in anterior cerebellum, closer to motor cerebellar regions, but reduced representation in posterior cerebellum. By contrast, the lateral subnetwork (Fig. 2*C*) was not represented in the striatum or thalamus, though it exhibited the most extensive cerebellar representation, anterior to other subnetworks in the anterior lobe and surrounding them in the posterior lobe. Finally, the Pars Marginalis subnetwork (Fig. 2*D*) exhibited no representation in the striatum or thalamus and only minimal representation in the cerebellum, in far anterior posterior lobe. See *SI Appendix*, Table S1 for coordinates of maximal cross-subject overlap within each subnetwork.

### Meta-Analytic Network Annotation (MANA) of AMN Subnetworks.

The functional role of each AMN subnetwork was determined by matching it to distributed activation patterns within the Neurosynth meta-analysis database (89). Meta-analytic imaging tools such as Neurosynth enable associations between specific brain locations reported as activated in task fMRI studies and textual terms extracted from the study manuscripts. Critically, these approaches cannot query terms associated with distributed network or subnetwork patterns consisting of multiple brain locations. Therefore, we developed MANA, a method for annotating brain networks. MANA expands the traditional meta-analytic approach, utilizing the Neurosynth database of brain coordinates and textual terms to consider distributed sets of activation peaks rather than isolated locations.

**Fig. 3.**
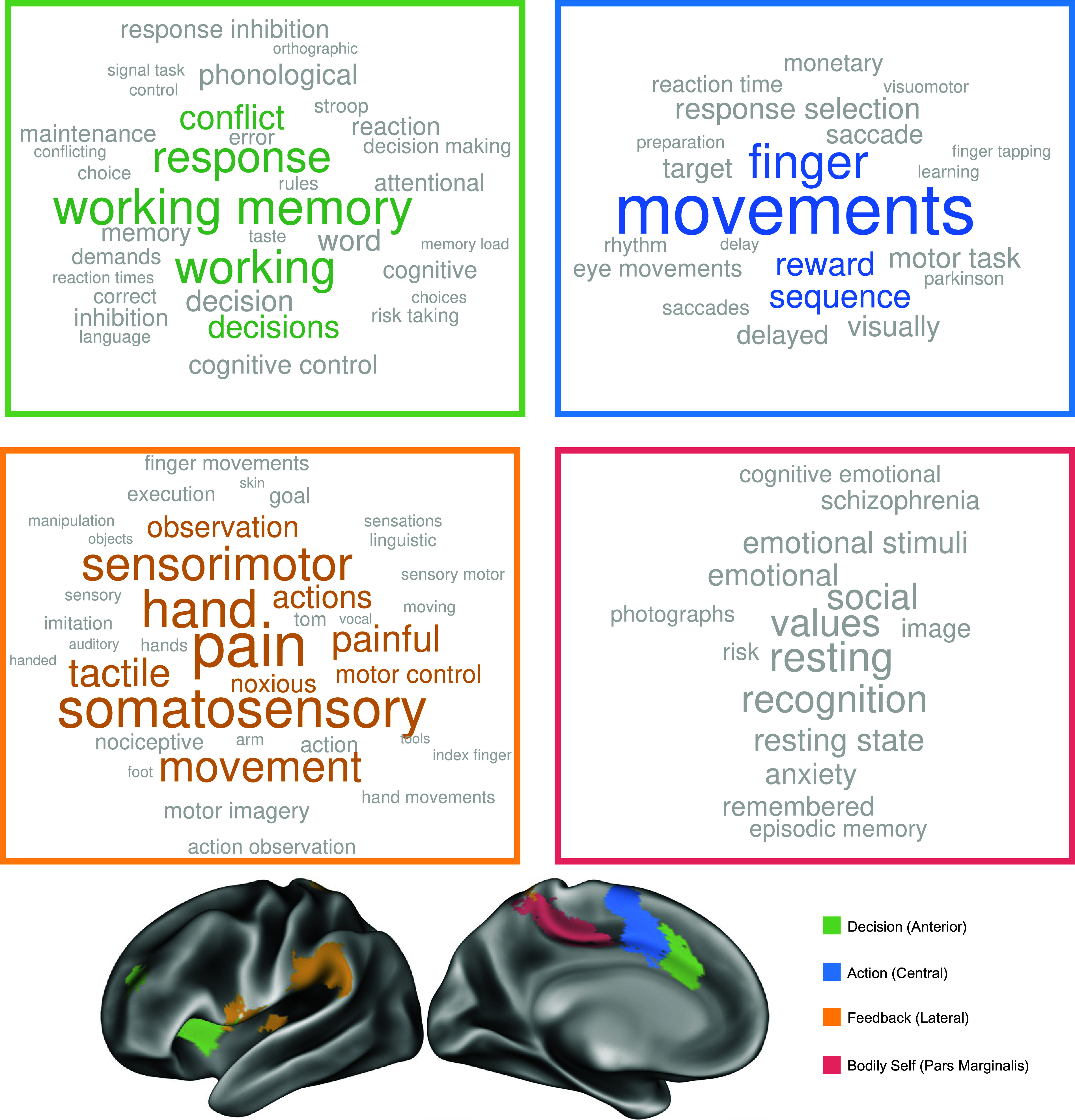
MANA of action-mode subnetworks. Subnetworks from the cross-participants winner-take-all analysis were matched to spatial activation distributions that had task descriptor terms in the Neurosynth database (89). Word clouds illustrate terms more associated with activation patterns of each action-mode subnetwork compared to the other subnetworks (tested for each term via one-way ANOVA). Larger font size indicates a higher frequency of the term. Terms shown in black are significant at *P* < 0.05 (unc.); colored terms are significant at *P* < 0.05, FDR corrected for the 742 terms tested.

For each AMN subnetwork, MANA identified studies reporting multiple activation peaks congruent with the spatial distribution of that subnetwork (Fig. 3; see *Methods*). For each database term, a one-way ANOVA tested whether the weighting of that term in those studies was significantly different across the three subnetworks. MANA word clouds illustrate the terms with significant differences, associated with the subnetwork for which it had the highest weighting. Word size is scaled to the magnitude of differences among subnetworks. Terms shown in black exhibited differences between subnetworks that were significant at *P* < 0.05 (unc.); colored terms were significant at *P* < 0.05, FDR corrected for the total number of terms tested.

The terms “working memory,” “response,” “conflict,” and “decisions” were relatively most strongly associated with the anterior subnetwork (*P* < 0.05, FDR). Together, in the context of the overall AMN function of selecting and implementing actions, these terms suggest a circuit that engages complex cognitive processes that integrate information from multiple sources, including resolving conflicts among conflicting sources, to decide on a course of action. Thus, the anterior AMN was functionally annotated as the Decision subnetwork (green).

Premotor terms such as “sequence,” and pure motor terms such as “movements,” and “finger” were most strongly associated with the central AMN subnetwork. The central subnetwork also included the term “reward.” These terms suggest a circuit that generates an action plan. Thus, the central AMN was functionally annotated as the Action subnetwork (blue).

The lateral AMN subnetwork was associated with nociceptive terms such as “pain,” “painful,” and “noxious,” and with somatosensory terms such as “somatosensory,” “sensorimotor,” and “tactile,” but also with “observation” and (nonsignificantly) “action observation,” “motor imagery,” and “imitation.” Generally, these terms suggest a circuit that processes multiple feedback modalities critical for actions, such as pain and sensory/visual action feedback. Thus, the lateral AMN was functionally annotated as the Feedback subnetwork (yellow).

Notably, the Pars Marginalis AMN subnetwork was not significantly associated with any terms. We hypothesized that this could be caused by a paucity of extant studies eliciting activation within this region. To test this hypothesis, we mapped all reported study activations within the Neurosynth database to the cortical surface. We observed that the pars marginalis of the cingulate sulcus exhibited some of the fewest reported activations of any brain region outside of locations associated with susceptibility artifact (*SI Appendix*, Fig. S2). This suggests that the function of the Pars Marginalis AMN subnetwork may not yet be clearly identifiable using extant task-based fMRI paradigms. However, recent human electrophysiological studies of regions in the posteromedial cortex reported that the Pars Marginalis of the cingulate may serve as a convergence zone for bodily schema, and to be critical for constructing the bodily self (91), something that would be difficult to capture with task fMRI (*Discussion*). Therefore, based on strong human direct stimulation evidence, we are tentatively labeling the pars marginalis subnetwork as the “Bodily Self,” though this functional label may be subject to future revision.

We note that the MANA approach identifies which subnetwork is most strongly associated with each term, but this must not be misinterpreted as an absence of association for other subnetworks. For example, pain is most strongly associated with the Feedback subnetwork, but it is also associated with the Decision and Action subnetworks of the AMN.

To confirm differences in evoked activity between AMN subnetworks, we examined task data collected in 10 of the 15 participants across five motor task conditions [motor task (92): Tongue, Left Hand, Right Hand, Left Leg, Right Leg] and two cognitive conditions [spatial, verbal discrimination (2)]. See *SI Appendix*, Fig. S3.

For each condition, we entered subnetwork activation values into an ANOVA model testing for main effects of subnetwork, with subject identity modeled as a random effect, and Bonferroni-correcting for the number of networks tested. We observed significant main effects of subnetwork for all tasks tested [Fs(3, 26) > 14.0, *P*s < 0.001]. Post hoc paired *t* tests demonstrated that the AMN’s Decision and Action subnetworks were more active during Tongue movement than the Feedback or Bodily Self subnetworks [ts(9) > 4.6, *P*s < 0.002]. The Action subnetwork was more activated than any other subnetwork by all Hand and Foot movements [ts(9) > 2.53, *P*s < 0.04], except for the Right Hand movement [t(14) = 1.87, *P* < 0.10]. The Decision and Action subnetworks were more active than the Feedback or Bodily Self subnetworks during both of the cognitive conditions (spatial and verbal discrimination) [ts(9) > 4.31, *P*s < 0.002], though the Decision and Action subnetworks did not differ from each other. See *SI Appendix*, Table S4 for *t* tests for all subnetworks, for all tasks.

### AMN Subnetworks Exhibit Differential Connectivity with Other Functional Networks.

**Fig. 4.**
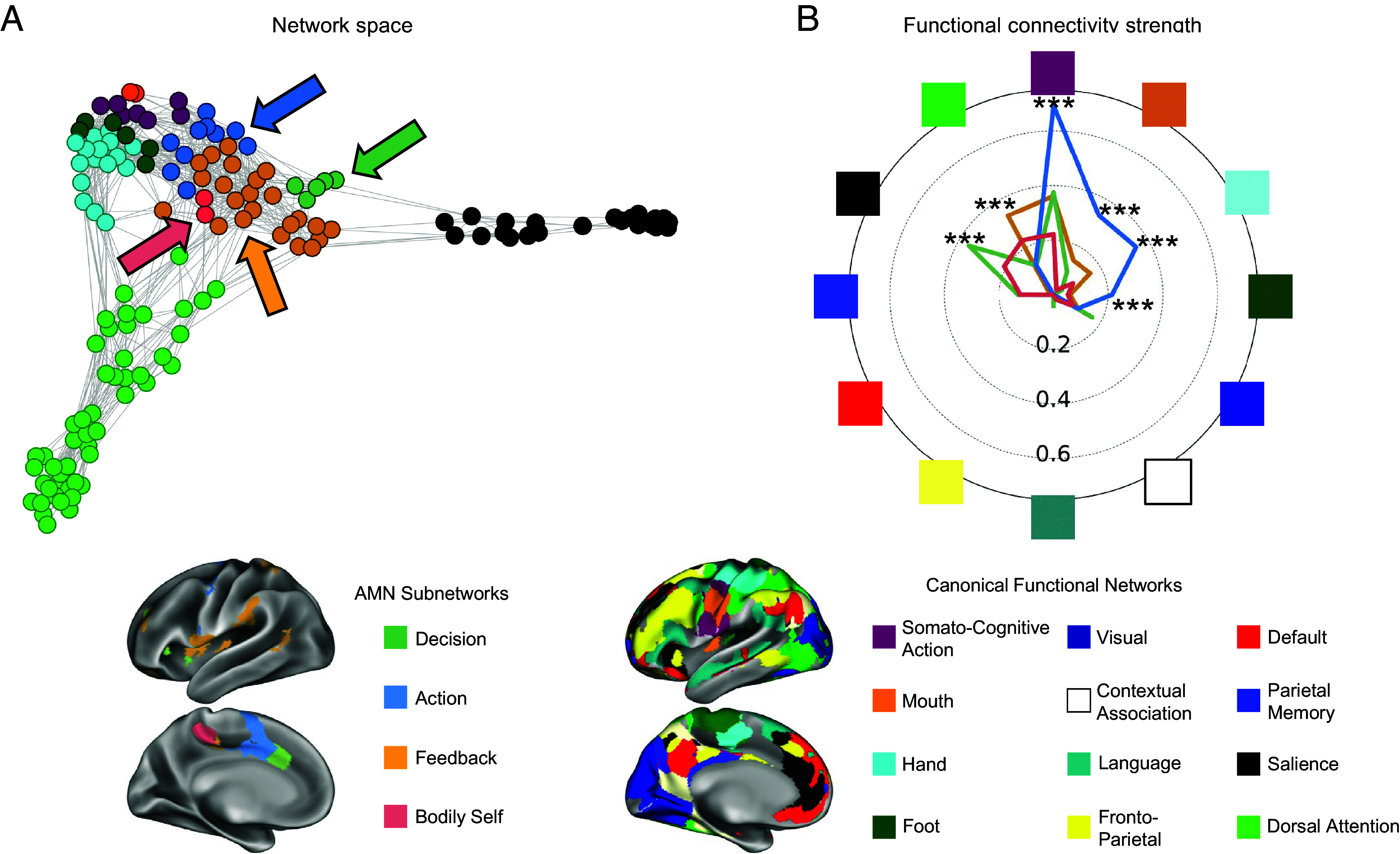
Functional connectivity patterns of AMN subnetworks. (*A*) A spring-embedded plot in an exemplar participant (P01) illustrates the preferential connectivity of Action Mode subnetworks to large-scale functional networks. For clarity of visualization, only networks most closely associated with the subnetworks are shown. See *SI Appendix*, Fig. S4 for all individual participants. (*B*) Across participants, individual-specific action-mode subnetworks demonstrate preferential connectivity to other individual-specific functional networks. The radial axis indicates the strength of functional connectivity Z(r) between each AMN subnetwork and each canonical functional network. Negative connectivity values are not represented. Significant differences among subnetwork connectivities to each network, determined via ANOVA, are indicated as ***P(corr.) < 0.001. Colors and spatial locations of AMN subnetworks (*Left*) and other canonical networks (*Right*) are shown in the exemplar participant at the *Bottom*.

Spring-embedded graph visualizations (following methods from refs. 5, 45, and 93); exemplar participant P01 shown in Fig. 4*A*; all participants in *SI Appendix*, Fig. S4) suggest that the Decision, Action, Feedback, and Bodily Self AMN subnetworks exhibit preferential connectivity to canonical functional networks. Quantification of average connectivity to each network (Fig. 4*B*) showed that the Decision subnetwork was most closely linked to the Salience network. This connectivity may enable reward and/or aversive signals generated by the Salience network to bias action decisions. The Action subnetwork was most closely linked to the SCAN, with additional strong connectivity to effector-specific (foot, hand, mouth) motor networks. This connectivity may enable transmission of the generated action plan to motor execution circuits. The Feedback subnetwork was most closely linked to the Dorsal attention network, which may allow the visual attention functions of DAN to contribute to action observation. The Bodily Self subnetwork was not strongly connected to any other large-scale network, suggesting that its function may be internal to the AMN.

To determine whether AMN subnetworks differed from each other in their functional connectivity to canonical functional networks, for each network, we entered subnetwork connectivity values into an ANOVA model testing for main effects of subnetwork, with subject identity modeled as a random effect. *P* values for each network-level ANOVA were Bonferroni-corrected for the number of networks tested. We observed significant main effects of subnetwork for the SCAN, DAN, Salience, Somatomotor-hand, Somatomotor-face, and Somatomotor-foot [Fs(3, 38) > 7.4, *P*s < 0.001].

Post hoc paired *t* tests demonstrated that the Action subnetwork exhibited stronger connectivity than any other subnetwork to the SCAN, Somatomotor-hand, Somatomotor-face, and Somatomotor-foot networks [ts(12) > 3.7, *P*s < 0.004]. The Feedback subnetwork exhibited stronger connectivity than any other subnetwork to the Dorsal Attention network, though this difference was not significant vs. the Bodily Self subnetwork [Feedback vs. Action, vs. Decision: ts(12) > 4.2, *P*s < 0.001; Feedback vs. Bodily Self: t(12) = 2.0, *P* = 0.07]. Similarly, the Decision subnetwork exhibited stronger connectivity than any other subnetwork to the Salience network, though this difference was not significant vs. the Bodily Self subnetwork [Decision vs. Feedback, vs. Action: ts(12) = 6.5, *P*s < 0.001; Decision vs. Bodily Self: t(12) = 1.6, *P* = 0.14]. All post hoc paired *t* tests can be found in *SI Appendix*, Table S2.

Some of the strong subnetwork-to-network connectivity preferences (e.g., AMN-Decision to Salience) were between physically adjacent portions of the cortex. To ensure that the strong FC values were not driven by local autocorrelations of the fMRI signal, the above analyses were replicated after excluding all functional connections between cortical vertices within 20 mm of each other. Connectivity profiles did not change (*SI Appendix*, Fig. S5); all network-level ANOVAs remained significant (all *P*s < 0.005); and all post hoc *t* tests reported significant above remained significant (all *P*s < 0.005).

### Spontaneous fMRI Signals in AMN Subnetworks Are Temporally Ordered.

**Fig. 5.**
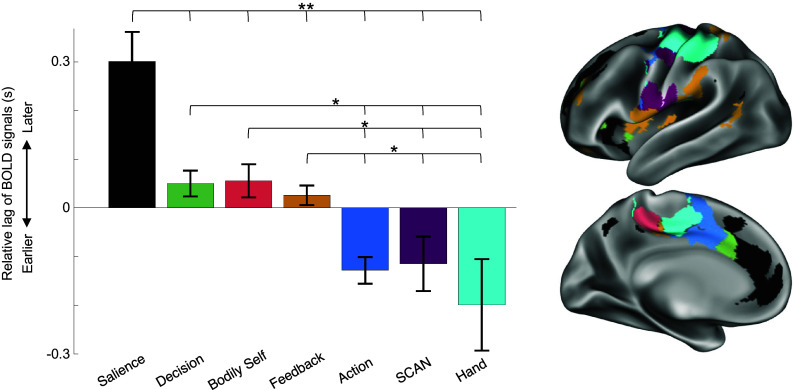
Temporal ordering of AMN signals in the action output processing stream. Temporal ordering of rs-fMRI signals for each individual-specific AMN subnetwork, as well as for the associated Salience, SCAN, and Hand networks. Values are averaged across vertices within each subnetwork and across participants. The zero-point is arbitrary and indicates timing relative to all AMN subnetworks. SE across participants is indicated by error bars. A one-way ANOVA indicated a significant main effect of subnetwork/network identity (*P* < 0.0001). ** indicates *P* < 0.01, * indicates *P* < 0.05 for all post hoc paired *t* tests. *Inset* shows subnetwork and network topography for the exemplar participant (P01). Prior electrophysiology work suggests that later infraslow (0.08 to 0.1 Hz) activity (here, the Salience network and AMN-Feedback, Decision, and Bodily Self subnetworks) corresponds to earlier delta-band (0.5 to 4 Hz) activity (94).

Differential timing of infraslow rs-fMRI signals (0.08 to 0.1 Hz) among network structures suggests a temporally ordered processing stream, as has been identified among different subnetworks of the default-mode network and among networks in the precentral gyrus (45, 73). Here, lag analyses demonstrate the temporal order of infraslow rs-fMRI signals within networks of the putative action output processing stream, including Salience, AMN subnetworks, SCAN, and effector-specific motor networks (Fig. 5). A one-way ANOVA found a significant main effect of subnetwork/network identity [F(6, 94) = 9.92, *P* < 0.0001]. Post hoc *t* tests indicated that signals in the Salience network occurred later than those in all AMN subnetworks, SCAN, or motor Hand [all ts(14) > 3.1, all *P*s < 0.009]. Further, spontaneous activity in Feedback, Decision, and Bodily Self subnetworks occurred later than Action, SCAN, or Hand activity [ts(14) > 2.3, all *P*s < 0.04]. All temporal lag *t* tests can be found in *SI Appendix*, Table S3. Previous reports have associated interregional lags in infraslow (<0.1 Hz) signals with temporally ordered delta activity (0.5 to 4 Hz) lags in the opposite direction (94). Thus, these results suggest a temporal ordering of delta-frequency activity from Salience to the AMN-Feedback, Decision, and Bodily Self subnetworks, and then to the Action subnetworks, SCAN, and effector-specific motor networks, consistent with the expected relative ordering of a feedback–decision–action cycle.

## Discussion

### A Functional Processing Stream of the AMN.

Traditional outside-in approaches for identifying brain regions associated with a priori cognitive functions have described a surprisingly varied range of functionality for AMN regions. Here, we employ an alternative, inside-out network annotation approach (59) to first identify the organization of the AMN and to subsequently probe function within this organization.

The brain-first, inside-out approach describes the AMN as a functionally integrated system with networked substructures that each have specific inputs and outputs, and which contain circuits spanning many cortical lobes and subcortical structures. These AMN subnetworks exhibit different task responses and sequential lagged timing of signals, suggesting that together they enable serial processing of information cutting across specific a priori psychological functions, and even across the traditional sensory/motor/cognitive domains.

We have argued that the AMN initiates and maintains the brain’s action mode (8), in which arousal is heightened, attention is externally focused, sympathetic input to internal organs drives physiological mechanisms such as respiration and heart rate, goals are established and maintained, decisions are made among potential competing outcomes, action plans are created and converted to movements, and feedback from action outcomes is evaluated to modify future decision and action plans. The AMN subnetwork organization described here constitutes the major cortico-subcortical processing stream within the action mode, enabling transformation of reward and motivational information into concrete action plans, and evaluating those actions to update future plans accordingly—the primary functions of the action mode.

Most AMN subnetworks were associated with specific cognitive functions (Fig. 3 and *SI Appendix*, Fig. S6). However, these subnetworks should not be thought of as performing a list of functions enumerated here. Rather, the inside-out framework argues that each subnetwork performs a specific type of processing that is employed during the execution of a variety of common fMRI tasks.

### An AMN-Decision Subnetwork for Action Selection, Maintenance, and Control.

The AMN’s Decision subnetwork described here overlaps with the functional core of the classic description of AMN (9–11), which is centered in bilateral dACC, anterior insula, and aPFC. The preferentially cognitive nature of functions associated with the Decision subnetwork also converges with previous reports of AMN functionality, including involvement in task maintenance, error processing, conflict monitoring, and ambiguity processing to decide among competing options (10, 33, 36–38, 95). Lesions of the AMN-Decision subnetwork cause apathy and abulia (24, 25), as well as decreased spontaneous self-initiated activity (96).

The AMN-Decision subnetwork was most strongly connected to the Salience network (97), which enables reward valuation and motivation (18); degeneration of Salience regions induces apathy (17). DACC regions in the Decision subnetwork evaluate reward information in order to make decisions (98), guide behavior (99, 100), and produce actions (101). Thus, Salience network may provide inputs to the AMN-Decision subnetwork enabling it to select decisions, exert control, and produce actions (via projections to the AMN-Action subnetwork) that are guided by the reinforcement value of that decision. The AMN-Decision subnetwork may also enable control over nonphysical actions, including working memory and attention functions.

### An AMN-Action Subnetwork for Initiation of and Control Over Physical Actions.

The AMN’s Action subnetwork converges with regions that initiate and maintain control over physical actions (motor outputs). Action initiation and control can be understood as a cascade of executive functions that proceed from abstract to concrete action plans, and then to execution of movement (102). The regions at one end of this processing stream—cingulate motor zones in the macaque dorsal medial prefrontal cortex—initiate a chain of projections backward to supplementary motor area, premotor area, and to motor cortex (48, 49). These cingulate motor zones appear spatially homologous with the AMN-Action subnetwork, which similarly exhibited strong connectivity with motor networks and strong activation during motor behavior.

The AMN-Action subnetwork was strongly connected to the recently identified SCAN, which implements actions on a whole-body level, including regulation of posture and internal physiology (45). The AMN-Action subnetwork further had representation in dorsal anterior putamen, which is associated with motor planning and learning and receiving projections from premotor areas in nonhuman primates (103). The Action subnetwork may receive fast somatosensory feedback about the results of executed actions, as AMN-Action regions were proximal to primary somatosensory cortex (S1) and secondary somatosensory cortex (S2) in the dorsal posterior insula, and MANA indicated somatosensory functions in the Action subnetwork. The Action subnetwork may also contribute to visceral organ control, which is localized in the posterior insula (104).

### An AMN-Feedback Subnetwork for Evaluating Action Outcomes.

The AMN-Feedback subnetwork likely provides temporally extended feedback to the Decision subnetwork based on the outcomes of prior actions. Unlike the Decision and Action subnetworks, the AMN-Feedback subnetwork exhibited little striatal or thalamic representation. Because cortico–striato–thalamo–cortical loops are critical for the motivation, development, planning, and execution of goal-directed actions (103), the absence of such loops in the Feedback subnetwork suggests that this circuit is not directly involved in the feedforward processing stream of action production and control. Consistent with this interpretation, the Feedback subnetwork did not activate during motor or cognitive tasks but was associated with meta-analytic terms reflecting somatosensory feedback, including processing of painful and noxious stimuli. The subnetwork’s representation in middle insula and SMG is consistent with known distributions of pain-related brain activity (51, 56); indeed, a large meta-analysis identified middle insula and SMG—the most prominent AMN-Feedback regions—as the most pain-associated regions of the brain (55). This suggests a role for the AMN, and specifically the Feedback subnetwork, in attending to, integrating, and transmitting sensory information, and particularly information related to pain, perhaps the most salient type of action-feedback (105).

In addition to somatosensation and pain, the Feedback subnetwork was associated with terms reflecting observation of actions; and further, it exhibited uniquely strong connectivity to the Dorsal Attention Network, which directs eye movements and processes attention-directed visuospatial information (106, 107). Thus, we hypothesize that the Feedback subnetwork processes multiple types of postaction feedback, including pain and relevant visual information about the results of an executed action. Such feedback mechanisms may be critical for accurate predictive modeling of future motor actions, which has been argued to involve the mid-insula (44). By contrast, the AMN-Feedback subnetwork may not be primary in processing another type of feedback, task errors (i.e., incorrect choices), as the “error” term was associated more strongly with the AMN-Decision subnetwork. Error-related signals are strongest in Decision subnetwork regions such as dACC and anterior insula but can also be observed in Feedback regions such as inferior parietal and inferior frontal gyri (9, 32, 33). Task errors, being more context-dependent than pain or visual feedback, may require adjudication by the Decision subnetwork.

The anterior-to-posterior anatomical position of the AMN-Decision and -Feedback subnetworks in the insula seems to converge with the known dysgranular-to-granular architectonic organization of the insula (108). Overlapping AMN subnetworks with architectonic divisions of the insula (109) revealed that the Decision subnetwork was only present in dysgranular insula divisions, while the Feedback subnetwork overlapped posterior granular insula divisions (*SI Appendix*, Fig. S6). Prior work suggests that the dysgranular division is more related to goal-directed, motivated behavior, while the granular division is related to sensorimotor integration (110), a distinction consistent with the present work.

### A Subnetwork in the Pars Marginalis for Integrating Sensation and Action into the Bodily Self.

The AMN subnetwork centered in the Pars Marginalis of the cingulate sulcus may be critical for constructing the sense of the bodily self. This subnetwork was notably difficult to associate with known function, given its lack of coverage in the Neurosynth database (*SI Appendix*, Fig. S2) and its lack of strong connectivity to other known brain networks. However, recent work using direct stimulation of regions in the posteromedial cortex reported that stimulation of the Pars Marginalis of the cingulate, but not of other surrounding regions, consistently induced feelings of floating and being disconnected from the body (91). Based on these observations, this region was argued to function as a convergence zone for bodily schema that is continually updated as an individual acts in the environment, and thus to be critical for constructing the bodily self (91). Network analyses revealed this bodily self-related region in the Pars Marginalis to be connected to the AMN (91), strongly suggesting convergence with the Pars Marginalis subnetwork described in the present work. As such, we tentatively label this subnetwork as AMN-Bodily Self, though this functional labeling must be considered subject to revision based on future work that may identify alternate or more extensive functions of this circuit.

### From Goal Establishment to Action Execution across AMN Subnetworks.

Relative differences in spontaneous rs-fMRI activity timing among the AMN subnetworks suggest a temporal sequence of activity from Salience to AMN-Feedback, -Decision, and -Bodily Self to AMN-Action, and then to the SCAN and effector-specific motor networks. Infraslow rs-fMRI signals (<0.1 Hz) were detected relatively later in Salience, Feedback, Bodily Self, and Decision AMN than in the Action AMN subnetwork. However, the “sender–receiver” model argues that the temporal ordering of infraslow activity (<0.1 Hz) is in the opposite direction from the temporal ordering of delta-band frequency (0.5 to 4 Hz) cortical activity reflective of neural processing, as the infraslow activity coordinates timing of information exchange via phase-amplitude coupling (94, 111). This result expands findings from our prior work, which described an AMN->SCAN->M1 ordering of signals (45). Here, the distinct ordering of spontaneous activity waves we observed within and beyond AMN provide important convergence with prior work conducting invasive recordings in humans and macaques. In humans, the posterior medial frontal cortex initiated activity preceding a task-driven response later than all other prefrontal, parietal, or temporal regions (112), similar to the Action AMN region described here. In macaques, signals in more rostral cingulate regions occur earlier before a movement than those in caudal cingulate, which in turn precede motor cortex activity (113). The Decision and Action portions of dorsal anterior cingulate may thus represent human homologs of the macaque rostral and caudal cingulate motor areas, respectively (48). These orderings further suggest that the strong connectivity between the Decision AMN and the Salience network may reflect the projection of inputs from Salience to the AMN.

Together, these findings suggest a processing stream of goal and action control and feedback adjustment in the brain. In this model, the AMN-Decision subnetwork receives connections reflecting valuations from regions in the Salience network that process reward (114) and incorporates this information to perform judgments, adjudicate between alternatives, to initiate and maintain goals (115). If a goal can be expressed as a physical action, it is projected to the Action subnetwork where the abstract goal is transformed into concrete action plans (48). Action plans are projected to the SCAN to prepare the whole body to implement the plan (45), and finally to effector-specific M1 where it is transformed into fine motor plans. Feedback about prior action outcomes (including salient visual stimuli and pain) is provided through the AMN-Feedback subnetwork to 1) the Decision subnetwork, which processes these signals, as well as goal-incompatible errors, to update active goals, and 2) to the Action subnetwork to enable predictive modeling of future planned actions (44). Information from all other subnetworks may be integrated into the Pars Marginalis-centered subnetwork for the construction of the Bodily Self.

The temporal ordering of spontaneous signals within the AMN, even in the absence of any goal-directed activity, likely reflect cyclic arousal-related global waves, as has been observed with fMRI in humans (116) and with fast optical imaging in mice (117). AMN sits at one end of this arousal-locked cycle, opposite the DMN (8, 116). The present observation that within AMN, these spontaneous signals exhibit temporally ordered activity along circuits convergent with a known action-to-motor hierarchy (113) suggests that in detail, arousal-driven signal fluctuations may be temporally sequenced along functional processing streams.

### Targeted Treatments of Patients with AMN Disruptions.

Understanding the organization and function of the AMN is of critical importance, as AMN regions are being investigated as targets for neuromodulation in the treatment of pain, addiction, epilepsy, and movement disorders (118–124). Patient-specific AMN subnetwork mapping can improve the characterization and treatment of neurological and psychiatric conditions affected by AMN processing. For example, lesions within AMN cause apathy and abulia (24, 25), decreasing spontaneous self-initiated activity. Abulia is also a key psychiatric symptom of Parkinson’s disease (125). Such deficits in activity may be most specifically related to damage to or deficits in the AMN-Decision subnetwork, such that goals leading to action are not set. Other Parkinson’s disease deficits such as freezing of gait represent an impaired ability to initiate intended movements (126, 127) and may be more specifically related to the AMN-Action subnetwork and its connectivity to SCAN, which enables whole-body actions. By contrast, chronic pain symptoms may be most related to the AMN-Feedback subnetwork, as AMN-Feedback regions (insula, SMG/inferior parietal lobule) were more associated with pain than other AMN regions in a large meta-analysis (55). Notably, the ability to delineate these disease-specific AMN subnetworks in individuals opens the door for neuromodulation therapies precisely targeting these brain regions.

### An Integrated Processing Stream with Ancient Evolutionary Precursors.

Cognitive neuroscience-derived models of brain organization have often employed the outside-in approach, and so must, by their nature, describe separate brain modules for each a priori cognitive/behavioral function tested. This has led to the conceptualization that decision-making (prefrontal), action and movement control (SMA/M1), and pain processing (insula) are wholly distinct systems. By contrast, the inside-out approach describes these functions as an integrated processing stream within a single overarching cortico–striatal–thalamo–cerebellar functional network that works together to implement control over behavior under the action mode. From this perspective, we hypothesize that the Salience–AMN–SCAN axis reflects a reward–goal–action–movement processing stream that has ancient evolutionary precursors, useful for even simple organisms that can sense nutrient concentrations and move toward them (58, 128). As evolution progressed, this processing stream became more elaborated and interconnected with brain systems enabling complex cognitive functions and movement outcomes, but it still retained its core function of producing actions. This is why lesions to core AMN regions eliminate voluntary actions while broadly preserving other functionality (24, 25).

## Methods

### Data.

Data for this project were compiled from three preexisting datasets comprising 15 PFM subjects with between 98 and 356 min of per-subject resting-state fMRI. These datasets are termed the Plasticity dataset (46), the Midnight Scan Club (MSC) dataset (2), and the Multi-Echo dataset (129). For details about participants, data acquisition, and processing, see the *SI Appendix*, *Supplemental Methods*.

### Analysis.

#### Mapping network structure.

The network organization of each participant’s brain was delineated following (16, 73) using the Infomap algorithm for community detection (130). This approach, which has the advantage over other algorithms of deriving networks only from the strongest positive connectivity values in the brain, has been extensively utilized in prior work for network identification and discovery (2, 5, 12, 13, 16, 45–47, 73, 84, 86, 93). Communities derived from sparse thresholds in this approach represent subnetworks, while communities derived from denser thresholds represent more traditional large-scale brain networks (5, 73), including AMN. For details of these approaches, see the *SI Appendix*, *Supplemental Methods*.

#### Matching AMN subnetworks across individuals.

For each participant, we considered subnetworks as communities defined at the 0.1% density level (i.e., each column and row in the connectivity matrix retains the top 0.1% of connectivity values). Previous validation of this procedure (73) has shown that the 0.1% density threshold identifies subnetwork divisions in resting data that best explain task activations in these participants. We identified these subnetworks that had representation within each subject’s individual-specific Action Mode network (defined above), with representation in the insula, anterior cingulate cortex extending dorsally into dorsomedial prefrontal cortex, supramarginal gyrus, and pars marginalis of the cingulate (2, 5, 6, 9–11).

We sorted all subjects’ AMN subnetworks into distinct clusters. This was accomplished by first computing the pairwise spatial overlap between subnetworks as their Jaccard coefficient, resulting in a # subnetworks X # subnetworks overlap matrix. This overlap matrix was fed into an iterative Louvain community detection algorithm (obtained from https://github.com/neuro-data-science/neuro_data_science/tree/master/matlab/basset_connectivity) which iteratively computed clusters until the modularity of the resulting clusters within the overlap matrix was no longer higher than the modularity of the previous set of clusters. Clusters of overlapping subnetworks present in fewer than half of subjects were not considered.

After identifying AMN subnetworks on the cortex, we examined subcortical areas (basal ganglia, thalamus, and cerebellum) for representation of our selected AMN subnetworks in these regions.

#### Visualizing subnetwork overlap across participants.

For each matched subnetwork, the number of individuals with each subnetwork present was calculated for each cortical vertex. For subcortical voxels, some participants (Plasticity and MSC) were in Talairach space. To represent overlap, we first linearly transformed each of these participants into MNI152 space using a standard Talairach-to-MNI linear transform. Overlap was then calculated across MNI-space participant subnetworks as the number of individuals with the subnetwork present in each MNI-space voxel. This procedure produced maps of the density of each subnetwork across participants.

#### MANA analysis.

We investigated functions associated with each subnetwork by leveraging text-mining and meta-analyses techniques in the brain mapping Neurosynth database (https://github.com/neurosynth/neurosynth-data). Neurosynth compiles neuroimaging studies and generates probabilistic mappings based on article term frequencies and term-to-activation correlations (89). Neurosynth functionality natively includes mapping single MNI152 [X, Y, Z] coordinates to meta-analytic descriptor terms common in the neuroimaging literature. This is accomplished by automatically text-mining both activity peaks and descriptor terms from a huge corpus of neuroimaging papers. Then, papers can be identified in which a reported activity peak falls within a short distance of the target coordinate. At the time the Neurosynth database was downloaded, it contained over 500,000 activation peaks from over 14,000 fMRI papers. Each paper is labeled with at least one term automatically mined from its text, and each term has a weighting for each paper reflecting the prevalence of that term in the text.

Here, we expanded this functionality by mapping not just a single coordinate, but instead simultaneously mapping the multiple regions within each subnetwork. We first identified the congruent clusters of each subnetwork present in the group. This was accomplished by thresholding the subnetwork density maps (representing cross-participant overlap, described above) to retain all cortical vertices in which >10% of participants had the subnetwork. For each subnetwork, we then identified all studies within the Neurosynth database with a collection of activation peaks that “matched” the subnetwork. Specifically, the study had to report an activation peak <2 mm from at least 40% of subnetwork regions. Thus, a given study must have elicited activity near a substantial number of the subnetwork’s regions in order to match that subnetwork. Varying these parameters did not substantially alter findings reported here.

All terms associated with matching studies that were related to mental or task-related function (e.g., “effort,” “oddball,” “language,” “delay,” “covert”), were retained, while all terms related to aspects of the participant population (e.g., “male”), brain location (e.g., “occipital,” “network”), or anything unrelated (e.g., “voxel,” “peripheral,” “extra”) were excluded. This restricted the database to 742 terms.

Terms in the Neurosynth database have “weights” for each study ranging from 0 to 1, indicating the prevalence of that term within the text-mined paper. For each term, we tested whether that term’s weights differed significantly across different subnetworks. Specifically, the weights found for that term in each study matched to each subnetwork were all entered into a one-way ANOVA testing for effects of the identity of the matching subnetwork. Significance in this test indicates significant differences in the weight of that term across subnetworks. If the ANOVA was significant, that term was associated with the subnetwork exhibiting the largest average weight. Significance was tested both at *P* < 0.05, FDR-corrected for the number of terms tested, as well as (for exploratory purposes) at *P* < 0.05 uncorrected.

#### Neurosynth activation peak density.

For each cortical vertex in the MNI152-space Conte69 atlas midthickness cortical surface, we computed the number of activation peaks within the MNI152-space Neurosynth database that were within 4 mm of that vertex.

#### Task analysis.

Modeling of task-evoked activity in this data has been previously described in ref. 2 and is fully described in *SI Appendix*, *Supplemental Methods*. The task contrasts of interest included: 1) Tongue > baseline, 2) Left Hand > baseline, 3) Right Hand > baseline, 4) Left Leg > baseline, 5) Right Leg > baseline, 6) Spatial Discrimination Task > baseline, 7) Verbal Discrimination Task > baseline. For each of these conditions, we calculated the average activation (z-scores) within the vertices of each subnetwork.

For each task condition, we conducted an ANOVA testing for main effects of subnetwork identity on activation in that condition, with subject identity entered as a random effect. *P* values for these main effects were Bonferroni-corrected for the number of task conditions tested. When significant main effects of subnetwork were identified, we followed up with post hoc subnetwork vs. subnetwork paired *t* tests.

#### Calculating connectivity to other networks/subnetwork–network relationships.

For each AMN subnetwork matched across participants, we calculated the average fMRI time course across subnetwork voxels/vertices. We then calculated the average time course across voxels/vertices of the large-scale Default Mode, Visual, Fronto-Parietal, Dorsal Attention, Language, Salience, Somatomotor Hand, Somatomotor Face, Somatomotor Foot, Parietal Memory, Context, and Somato-Cognitive Action networks. Voxels/vertices that overlapped with that subnetwork were excluded from these averages. We then calculated the functional connectivity between each AMN subnetwork and each of the 12 large-scale networks as the Fisher-transformed correlation of the two-time courses.

For each large-scale network, we conducted an ANOVA testing for main effects of subnetwork identity on strength of connectivity to that network, with subject identity entered as a random effect. *P* values for these main effects were Bonferroni-corrected for the number of networks tested. When significant main effects of subnetwork were identified, we followed up with post hoc subnetwork vs. subnetwork paired *t* tests.

Visualization of subnetwork–network relationships in individual participants was conducted using spring-embedded plots (5), as implemented in Gephi (https://gephi.org/). In each participant, nodes were defined as contiguous cortical subnetwork or network clusters larger than 20 mm^2^ from each matched AMN subnetworks, as well as from within the other large-scale networks shown to be strongly connected to AMN subnetworks. Pairwise connectivity between nodes was calculated as the Z-transformed correlation of their mean time courses. For visualization purposes, graphs were constructed by thresholding node-to-node connectivity matrices at 10% density. These graphs were then imported into Gephi. For three participants (P05, P07, and P13), whole networks (always either Salience or Dorsal Attention) were disconnected from the rest of the graph at this density threshold. Since the goal was to visualize connectivity with these networks, in these cases, we systematically increased the density threshold in 5% increments until all networks were connected to the graph. For these three participants, the graph became connected and was visualized at 45%, 30%, and 20% densities, respectively.

#### Calculating subnetwork time delays.

We computed lagged correlation estimates by extending a previously published method (131) to create a “lag map” for each AMN subnetwork in each subject representing the time delay of each vertex relative to the subnetwork. For details of this approach, see *SI Appendix*, *Supplemental Methods*. Lag maps values were averaged across all vertices within each network/subnetwork of interest and then across the four subnetwork-seeded lag maps within each subject. This produced an average time shift for each network/subnetwork within each subject relative to all other tested networks/subnetworks. A one-way ANOVA tested whether there were any differences in this average time shift across the subnetworks and networks of interest. Post hoc *t* tests determined the relative temporal ordering of networks and subnetworks.

#### Comparison of subnetworks to architectonics.

We downloaded the Julich Brain Cytoarchitectonic Atlas v2.0 (109) from (https://julich-brain-atlas.de/atlas). This volumetric atlas is in the space of the MNI152 ICBM2009C nonlinear asymmetric brain. To map this atlas to the cortical surface, we downloaded an existing Freesurfer reconstruction of this brain from (https://figshare.com/articles/dataset/FreeSurfer_reconstruction_of_the_MNI152_ICBM2009c_asymmetrical_non-linear_atlas/4223811). For optimal comparison with fMRI data, these Freesurfer-derived surfaces were transformed into 32k fs_LR space, and the Julich atlas was sampled into this 32k fs_LR space using the enclosing voxel sampling procedure available in Connectome Workbench 1.0 (132). Borders of these surface-mapped architectonic areas were generated using Connectome Workbench, and these borders were superimposed over the group-averaged AMN subnetworks.

## Supplementary Material

Appendix 01 (PDF)

## Data Availability

Data processing code for Plasticity and Midnight Scan Club subjects can be found here: https://gitlab.com/DosenbachGreene/ (133). Data processing code for Multi-Echo subjects can be found here: https://github.com/cjl2007/Liston-Laboratory-MultiEchofMRI-Pipeline (134). Additional analysis code used for this manuscript can be found here: https://gitlab.com/DosenbachGreene/SCAN/ (135). Software packages incorporated into the above pipelines for data analysis included: Matlab R2020b, https://www.mathworks.com/ (136). Connectome Workbench 1.5, http://www.humanconnectome.org/software/connectome-workbench.html (137). Freesurfer v6.2, https://surfer.nmr.mgh.harvard.edu/ (138). FSL 6.0, https://fsl.fmrib.ox.ac.uk/fsl/fslwiki (139). 4dfp tools, https://4dfp.readthedocs.io/en/latest/ (140). Infomap, www.mapequation.org (141). Data from individual Plasticity Dataset subjects is available in the openneuro repository here: https://openneuro.org/datasets/ds002766/versions/3.0.0 (142). Data from individual Midnight Scan Club Dataset subjects is available in the openneuro repository here: https://openneuro.org/datasets/ds000224/versions/1.0.4 (143). Data from individual Multi-Echo Dataset subjects is available on reasonable request. They are not yet available through public databases because data collection is still ongoing.
