# Supplementary material for: Action-mode subnetworks for decision-making, action control, and feedback
Source: Proc Natl Acad Sci U S A. 2025 Jun 30;122(27):e2502021122. doi: 10.1073/pnas.2502021122 (PMC12260544; doi:10.1073/pnas.2502021122)
Supplement: Supplementary file 1 — Appendix 01 (PDF) [file pnas.2502021122.sapp.pdf]

PNAS

Supporting Information for

Action-mode subnetworks for decision making, action control, and feedback

Carolina Badke D'Andrea[1,2,3], Timothy O. Laumann[2], Dillan J. Newbold[4], Charles J. Lynch[5], Mohammad Hadji[1], Steven M. Nelson[6], Ashley N. Nielsen[2,7], Roselyne J. Chauvin[7], Samuel R. Krimmel[7], Abraham Z. Snyder[1,7], Scott Marek[1,2,8], Deanna J. Greene[3], Marcus E. Raichle[1,7,10,11,12], Nico U.F. Dosenbach[7,9-13], Evan M. Gordon[1]

1. Department of Radiology, Washington University School of Medicine, St. Louis, Missouri 63110, USA
2. Department of Psychiatry, Washington University School of Medicine, St. Louis, Missouri 63110, USA
3. Department of Cognitive Science, University of California San Diego, La Jolla, California 92093, USA
4. Department of Neurology, New York University Medical Center, New York, New York 10016, USA
5. Department of Psychiatry, Weill Cornell Medicine, New York, New York, 10065
6. Department of Pediatrics, University of Minnesota Medical School, Minneapolis, Minnesota 55455, USA
7. Department of Neurology, Washington University School of Medicine, St. Louis, Missouri 63110, USA
8. AI for Health Institute, Washington University School of Medicine, St. Louis, Missouri 63110, USA
9. Program in Occupational Therapy, Washington University School of Medicine, St. Louis, Missouri 63110, USA
10. Department of Biomedical Engineering, Washington University in St. Louis, St. Louis, Missouri 63130, USA
11. Department of Psychological and Brain Sciences, Washington University School of Medicine, St. Louis, Missouri 63110, USA
12. Department of Neuroscience, Washington University School of Medicine, St. Louis, Missouri 63110, USA
13. Department of Pediatrics, Washington University School of Medicine, St. Louis, Missouri 63110, USA

Corresponding Authors:
Evan Gordon, PhD
egordon@wustl.edu

Marcus Raichle, PhD
mraichle@wustl.edu

This PDF file includes:

Supporting text
Figures S1 to S7
Tables S1 to S4
SI References

Supporting Information Text

Supplemental Methods:

Dataset 1: Plasticity dataset

Participants:

Data were collected from 3 healthy, right-handed adult participants (ages 35, 25, and 27; one female; identified here as P01-P03) as part of a study investigating effects of arm immobilization on brain plasticity (data previously published in (1–4). Two participants are authors (NUFD and ANN). The remaining participant (male, 27 years old) was recruited from the Washington University community. Informed consent was obtained from all participants. The study was approved by the Washington University School of Medicine Human Studies Committee and Institutional Review Board. All data employed here was collected either prior to the immobilization intervention (Participants P02-03) or two years afterwards (Participant P01), and so we do not report details of that intervention.

MRI image acquisition:

Participants were scanned every day for twelve consecutive days using a Siemens Prisma 3T scanner on the Washington University Medical Campus. Every session included a 30-minute resting-state fMRI scan collected as a blood oxygen level-dependent (BOLD) contrast sensitive gradient echo-planar sequence (TE=33 ms, flip angle=84°, resolution=2.6 mm isotropic, TR=1100 ms, multiband 4 acceleration). During this scan, participants were instructed to hold still and look at a white fixation crosshair presented on a black background. A pair of spin echo EPI images with opposite phase encoding directions (AP and PA) but identical geometrical parameters to the BOLD data were acquired to correct spatial distortions.

For all fMRI data, head motion was tracked in real time using Framewise Integrated Real-time MRI Monitoring software (FIRMM (5)). An eye-tracking camera (EyeLink, Ottawa) was used to monitor participants for drowsiness.

For Participant 02 and 03, every session also included collection of a high-resolution T1-weighted MP-RAGE (TE=2.22ms, TR=2400ms, flip angle=8°, 208 slices with 0.8x0.8x0.8mm voxels) and a T2-weighted spin-echo image (TE=563ms, TR=3200ms, flip angle=120°, 208 slices with 0.8x0.8x0.8mm voxels).

For Participant 01, the structural images used were collected as part of Dataset 2 and consisted of four T1-weighted images (sagittal, 224 slices, 0.8 mm isotropic resolution, TE=3.74 ms, TR=2400 ms, TI=1000 ms, flip angle = 8°) and four T2-weighted images (sagittal, 224 slices, 0.8 mm isotropic resolution, TE=479 ms, TR=3200 ms) collected on a Siemens TRIO 3T scanner.

Dataset 2: Midnight Scan Club Dataset

Participants:

Data were collected from 10 healthy, right-handed, young adult participants (5 females; age: 24–34; identified here as P08-P15). The participants were recruited from the Washington University community. Other findings using these participants have been previously reported in (6–10). Two participants were excluded because they were also participants in the Plasticity dataset. One participant is an author (SMN). Informed consent was obtained from all participants. The study was approved by the Washington University School of Medicine Human Studies Committee and Institutional Review Board.

MRI image acquisition:

Imaging for each participant was performed on a Siemens TRIO 3T MRI scanner over the course of 12 sessions conducted on separate days, each beginning at midnight. Structural MRI was conducted across two separate days. In total, four T1-weighted images (sagittal, 224 slices, 0.8 mm isotropic resolution, TE=3.74 ms, TR=2400 ms, TI=1000 ms, flip angle = 8 degrees), four T2-weighted images (sagittal, 224 slices, 0.8 mm isotropic resolution, TE=479 ms, TR=3200 ms), four MRA (transverse, 0.6 x 0.6, x 1.0mm, 44 slices, TR=25ms, TE=3.34ms) and eight MRVs, including four in coronal and four in sagittal orientations (sagittal: 0.8 x 0.8 x 2.0 mm thickness, 120 slices, TR=27 ms, TE=7.05ms; coronal: 0.7 x 0.7 x 2.5 mm thickness, 128 slices, TR=28ms TE=7.18 ms), were obtained for each participant. Analyses of the MRA and MRV scans are not reported here. On ten subsequent days, each participant underwent 1.5 hours of functional MRI scanning beginning at midnight. In each session, we first collected thirty contiguous minutes of resting state fMRI data, in which participants visually fixated on a white crosshair presented against a black background. Each participant was then scanned during performance of three separate tasks: motor (2 runs per session, 7.8 minutes combined); mixed design including both a spatial and a verbal discrimination condition (2 runs per session, 14.2 minutes combined); and incidental memory (3 runs per session, 13.1 minutes combined). Preliminary analysis of the incidental memory task indicated that AMN subnetworks were not active during this task, so we do not report results in detail here. Across all sessions, each participant was scanned for 300 total minutes during the resting state and approximately 350 total minutes during task performance. All functional imaging was performed using a gradient-echo EPI sequence (TR=2.2 s, TE=27 ms, flip angle=90°, voxel size=4 mm x 4 mm x 4 mm, 36 slices). In each session, one gradient echo field map sequence was acquired with the same prescription as the functional images. An EyeLink 1000 eye-tracking system (http://www.sr-research.com) allowed continuous monitoring of participants' eyes in order to check for periods of prolonged eye closure, potentially indicating sleep. Only one participant (P13) demonstrated prolonged eye closures.

Task design:

Motor task design: The motor task was adapted from that used in the Human Connectome Project (11). Participants were presented with visual cues that directed them to close and relax their hands, flex and relax their toes, or wiggle their tongue. Each block started with a 2.2 s cue indicating which movement was to be made. After this cue, a centrally-presented caret replaced the instruction and flickered once every 1.1 s

(without temporal jittering). Each time the caret flickered, participants executed the proper movement. 12 movements were made per block. Each task run consisted of 2 blocks of each type of movement as well as 3 blocks of resting fixation, which lasted 15.4 s.

Mixed block/event-related design task: This task was adapted from experimental conditions reported by (12). One task was a spatial coherence discrimination task, which used concentric dot patterns (13) that were either 0% or 50% coherent. During this task, participants had to identify each pattern as concentric or random. The other task was a verbal discrimination task. Participants were presented with nouns and verbs and had to identify which type of word was being presented on the screen. Task blocks began with a 2.2 s cue screen indicating which task was to be conducted in the following block. Blocks consisted of 30 trials (half concentric/half nonconcentric for coherence, half noun/half verb for verbal). Stimuli were presented for 0.5 s with a variable 1.7-8.3 s ISI. A stop cue displayed for 2.2 s signaled the end of each task block. Each scan run consisted of two blocks of each task. Task blocks were separated by 44 s periods of rest. For each task, the finger used for each response was counterbalanced within participants across sessions.

MRI Processing: Dataset 1 & 2

Structural Processing:

Structural images (T1- and T2-weighted) were corrected for gain field inhomogeneity using FSL Fast (14) and aligned to the 711-2B implementation of Talairach atlas space using the 4dfp MRI processing software package (https://readthedocs.org/projects/4dfp/). The 711-2B template conforms to the 1988 Talairach atlas (15) according to the method of (16). Relative to MNI152, 711-2B space is about 5% smaller and 2° anteriorly rotated about the ear-to-ear axis. Mean T1- and T2-weighted images (T1w and T2w) were computed by coregistration and averaging multiple acquisitions.

Generation of cortical surfaces from the MRI data followed a procedure similar to that previously described in (8). First, anatomical surfaces were generated from the participant's average T1-weighted image in native volumetric space using FreeSurfer's default recon-all processing pipeline (version 5.3). This pipeline first conducted brain extraction and segmentation. After this step, segmentations were hand-edited to maximize accuracy. Subsequently, the remainder of the recon-all pipeline was conducted on the hand-edited segmentations, including generation of white matter and pial surfaces, inflation of the surfaces to a sphere, and surface shape-based spherical registration of the participant's original surface to the fsaverage surface (17, 18). The fsaverage-registered left and right hemisphere surfaces were brought into register with each other using deformation maps from a landmark-based registration of left and right fsaverage surfaces to a hybrid left-right fsaverage surface ('fs_LR'; (19)). These fs_LR spherical template meshes were input to a flexible Multi-modal Surface Matching (MSM) algorithm using sulcal features to register templates to the atlas mesh (20). These newly registered surfaces were then down-sampled to a 32,492 vertex surface (fs_LR 32k)

for each hemisphere. These various surfaces in native stereotaxic space were then transformed into atlas space (711-2B) by applying the previously calculated T1-to-atlas transformation.

<u>fMRI Preprocessing:</u>

Functional data were preprocessed to reduce artifacts and to maximize cross-session registration. All sessions underwent correction of odd vs. even slice intensity differences attributable to interleaved acquisition, intensity normalization to a whole brain mode value of 1000, and within run correction for head movement. Atlas transformation was computed by registering the mean intensity image from a single BOLD session to atlas space via the average high-resolution T2-weighted image and average high-resolution T1-weighted image. All subsequent BOLD sessions were linearly registered to this first session. This atlas transformation, mean field distortion correction (see below), and resampling to 3-mm isotropic atlas space were combined into a single interpolation using FSL's applywarp tool. All subsequent operations were performed on the atlas-transformed volumetric time series.

<u>Distortion Correction:</u>

A mean field map was generated based on the field maps collected in each participant (21). This mean field map was then linearly registered to each session and applied to that session for distortion correction. To generate the mean field map the following procedure was used: (1) 4 Field map magnitude images were mutually co-registered. (2) Transforms between all sessions were resolved. Transform resolution reconstructs the n-1 transforms between all images using the n(n-1)/2 computed transform pairs. (3) The resolved transforms were applied to generate a mean magnitude image. (4) The mean magnitude image was registered to an atlas representative template. (5) Individual session magnitude image to atlas space transforms were computed by composing the session-to-mean and mean-to-atlas transforms. (6) Phase images were then transformed to atlas space using the composed transforms, and a mean phase image in atlas space was computed. Application of mean field map to individual fMRI sessions: (1) For each session, field map uncorrected data were registered to atlas space, as above. (2) The generated transformation matrix was then inverted and applied to the mean field map to bring the mean field map into the session space. (3) The mean field map was used to correct distortion in each native-space run of resting state and task data in the session. (4) The undistorted data were then re-registered to atlas space. (5) This new transformation matrix and the mean field map then were applied together to resample each run of resting state and task data in the session to undistorted atlas space in a single step.

<u>RSFC Preprocessing:</u>

Additional preprocessing steps to reduce spurious variance unlikely to reflect neuronal activity were executed as recommended in (22, 23). First, temporal masks were created to flag motion-contaminated frames. Motion estimate time courses were filtered retain effects occurring below 0.1 Hz in order to eliminate "pseudomotion" induced by breathing-related motion of the chest altering the B0 field (24). Motion contaminated volumes were

then identified by frame-by-frame displacement (FD). Frames with FD > 0.2mm were flagged as motion-contaminated.

After computing the temporal masks for high motion frame censoring, the data were processed with the following steps: (i) demeaning and detrending, (ii) linear interpolation across censored frames using so that continuous data can be passed through (iii) a band-pass filter (0.005 Hz < f < 0.1 Hz) without re-introducing nuisance signals (25) or contaminating frames near high motion frames.

Next, the filtered BOLD time series underwent a component-based nuisance regression approach (8). Nuisance regression using time series extracted from white matter and cerebrospinal fluid (CSF) assumes that variance in such regions is unlikely to reflect neural activity. Variance in these regions is known to correspond largely to physiological noise (e.g., CSF pulsations), arterial pCO2-dependent changes in T2*-weighted intensity and motion artifact; this spurious variance is widely shared with regions of interest in gray matter. We also included the mean signal averaged over the whole brain as a nuisance regressor. Global signal regression (GSR) has been controversial. However, the available evidence indicates that GSR is a highly effective de-noising strategy (22, 26).

Nuisance regressors were extracted from white matter and ventricle masks, first segmented by FreeSurfer (27), then spatially resampled in register with the fMRI data. Voxels surrounding the edge of the brain are particularly susceptible to motion artifacts and CSF pulsations (28, 29); hence, a third nuisance mask was created for the extra-axial compartment by thresholding the temporal standard deviation image (SDt > 2.5%), excluding a dilated whole brain mask. Voxelwise nuisance time series were dimensionality reduced as in CompCor (30), except that the number of retained regressors, rather than being a fixed quantity, was determined, for each noise compartment, by orthogonalization of the covariance matrix and retaining components 5 ordered by decreasing eigenvalue up to a condition number of 30 (max eigenvalue / min eigenvalue > 30). The retained components across all compartments formed the columns of a design matrix, X, along with the global signal, its first derivative, and the six time series derived by retrospective motion correction. The columns of X are likely to exhibit substantial co-linearity. Therefore, to prevent numerical instability owing to rank-deficiency during nuisance regression, a second-level SVD was applied to XXT to impose an upper limit of 250 on the condition number. This final set of regressors was applied in a single step to the filtered, interpolated BOLD time series, with censored data ignored during beta estimation. Censored frames were then excised from the data for all subsequent analyses.

<u>Surface processing and CIFTI generation of BOLD data:</u>
Surface processing of BOLD data proceeded through the following steps. First, the BOLD fMRI volumetric timeseries (both resting-state and task) were sampled to each participant’s original mid-thickness left and right-hemisphere surfaces (generated as the average of the white and pial surfaces) using the ribbon-

constrained sampling procedure available in Connectome Workbench 1.0 (31). This procedure samples data from voxels within the gray matter ribbon (i.e., between the white and pial surfaces) that lie in a cylinder orthogonal to the local mid-thickness surface weighted by the extent to which the voxel falls within the ribbon. voxels with a timeseries coefficient of variation 0.5 standard deviations higher than the mean coefficient of variation of nearby voxels (within a 5 mm sigma Gaussian neighborhood) were excluded from the volume to surface sampling, as described in (32). Once sampled to the surface, timecourses were deformed and resampled from the individual's original surface to the 32k fs_LR surface in a single step using the deformation map generated above (in "Cortical surface generation"). This resampling allows point-to-point comparison between each individual registered to this surface space.

These surfaces were then combined with volumetric subcortical and cerebellar data into the CIFTI format using Connectome Workbench, creating full brain timecourses excluding nongray matter tissue. Subcortical (including accumbens, amygdala, caudate, hippocampus, pallidum, putamen, and thalamus) and cerebellar voxels were selected based on the FreeSurfer segmentation of the individual participant's native-space average T1, transformed into atlas space, and manually inspected. Finally, the BOLD timecourses were smoothed with a geodesic 2D (for surface data) or Euclidean 3D (for volumetric data) Gaussian kernel of σ = 2.55 mm.

Regression of adjacent cortical tissue from RSFC BOLD:

Many subcortical areas, such as dorsal cerebellum and lateral putamen, are in close anatomical proximity to cortex, resulting in spurious functional coupling between the cortical vertices and adjacent subcortical voxels. To reduce this artifact, RSFC BOLD time series from all vertices falling within 20mm Euclidean distance of a source voxel were averaged and then regressed from the voxel time series (8, 10, 33, 34). The resulting residual timeseries were used for all subsequent analyses.

Dataset 3: Multi-echo dataset

Participants:

Data were collected from 4 healthy, right-handed adults (0 female; ages 29, 39, 24, and 31; identified here as P04-P07). Other findings using these participants have been previously reported in (1, 35). Informed consent was obtained from all participants. The study was approved by the Weill Cornell School of Medicine Institutional Review Board.

MRI Acquisition:

Data were acquired on a Siemens Magnetom Prisma 3T scanner at the Citigroup Biomedical Imaging Center of Weill Cornell's medical campus using a Siemens 32-channel head coil. Either 24 (subjects 04, 05) or 12 (subjects 06, 07) fMRI runs were collected per subject. Each FMRI run consisted of a multi-echo, multi-band resting-state scans collected using a T2*-weighted echo-planar sequence covering the full brain (TR: 1355

ms; TE1: 13.40 ms, TE2: 31.11 ms, TE3: 48.82 ms, TE4: 66.53 ms, and TE5: 84.24 ms; FOV: 216 mm; flip angle: 68; 2.4mm isotropic; 72 slices; AP phase encoding direction; in-plane acceleration factor: 2; and multi-band acceleration factor: 6) with 640 volumes acquired per scan for a total per-run acquisition time of 14 min and 27 s, and a total per-subject scan time of 347 minutes (subjects 04, 05) or 173 minutes (subjects 06, 07). This sequence was generously provided by the Center for Magnetic Resonance Research (CMRR) at the University of Minnesota. A pair of spin echo EPI images with opposite phase encoding directions (AP and PA) but identical geometrical parameters and echo spacing were acquired to correct spatial distortions. High-resolution (MPRAGE) T1-weighted image (TR: 2400 ms; TE: 2.28 ms; FOV: 256; flip angle: 90, and 208 sagittal slices with a 0.8 mm thickness) and T2-weighted anatomical images (TR: 3200 ms; TE: 563 ms; FOV: 256; flip angle: 8, and 208 sagittal slices with a 0.8 mm thickness) were acquired. Custom headcases were obtained from Caseforge (https://ipira.berkeley.edu/caseforge-inc) for each participant to improve comfort and minimize head motion during scanning (36).

MRI Processing: Dataset 3

Cortical Surface Generation:

The average T1- and T2-weighted images were cropped to a smaller field of view (170mm in z plane), co-registered using FSL's epi_reg tool (via a boundary-based cost function with 6 DOF), and corrected for intensity inhomogeneities (37). The T1- and T2-weighted images were co-registered to an MNI atlas (hereafter referred to as "ACPC" alignment) using a rigid 6 DOF FLIRT transformation. Cortical surfaces were generated using Freesurfer's "recon-all.v6.hires" pipeline. Pial surface placement was refined using the co-registered T2-weighted image by specifying the "-T2pial" option. Midthickness surfaces were obtained by averaging the pial and white surfaces. Fsaverage-registered left and right hemisphere surfaces (pial, white, and midthickness) were brought into register with each other in fs_LR space (19) and resampled to the computationally tractable resolution of 32k vertices using Connectome Workbench command line utilities.

fMRI Preprocessing:

Preprocessing of multi-echo data minimized spatial interpolation and volumetric smoothing while preserving the alignment of echoes. The single-band reference (SBR) images (five total; one per echo) for each scan were averaged. The resultant average SBR images were aligned, averaged, co-registered to the ACPC aligned T1-weighted anatomical image, and simultaneously corrected for spatial distortions using FSL's topup and epi_reg programs. Freesurfer's bbregister algorithm (38) was used to refine this co-registration. For each scan, echoes were combined at each time point and a unique 6 DOF registration (one per volume) to the average SBR image was estimated using FSL's MCFLIRT tool (39) using a 4-stage (sinc) optimization. All of these steps (co-registration to the average SBR image, ACPC alignment, and correcting for spatial distortions) were concatenated using FSL's convertwarp tool and applied as a single spline warp to individual volumes of each echo after correcting for slice time differences using FSL's slicetimer program. All denoising was performed on these preprocessed, ACPC-aligned images.

Multi-echo denoising:

Multi-echo ICA (ME-ICA; (40, 41) denoising designed to isolate spatially structured T2∗- (neurobiological; "BOLD-like") and S0-dependent (non-neurobiological; "not BOLD-like") signals was performed using a modified version of the "tedana.py" workflow (https://tedana.readthedocs.io/en/latest/). In short, the preprocessed, ACPC-aligned echoes were first combined according to the average rate of T2∗ decay at each voxel across all time points by fitting the monoexponential decay, S(t) = S0e -t / T2∗, using the "nlinfit.m" function in MATLAB with least-squares optimization and the initial coefficient values obtained from a linear model fit to the log of the data. From these T2∗ values, an optimally combined multi-echo (OC-ME) time-series was obtained by combining echoes using a weighted average (WTE = TE ∗ e -TE/ T2∗), as in (42). The covariance structure of all voxel time-courses was used to identify major signals in the resultant OC-ME time-series using principal component and independent component analysis. Components were classified as either T2∗-dependent (and retained) or S0-dependent (and discarded), primarily according to their decay properties across echoes following the decision tree described in (40). Mean gray matter time-series regression was subsequently performed to remove spatially diffuse noise. Temporal masks were generated for censoring high motion time-points using a frame-wise displacement (FD; (43) threshold of 0.3 mm and a backward difference of two TRs (2 ∗ 1.355 = 2.77 s), for an effective sampling rate comparable to historical FD measurements (approximately 2 to 4 s; (36). Prior to the FD calculation, head realignment parameters were filtered using a stopband Butterworth filter (0.2 - 0.35 Hz) to attenuate the influence of respiration (36).

Surface processing and CIFTI generation of BOLD Data:

The denoised fMRI time-series was mapped to the midthickness surfaces (using the "-ribbon-constrained" method), combined into the Connectivity Informatics Technology Initiative (CIFTI) format, and spatially smoothed with geodesic (for surface data) and Euclidean (for volumetric data) Gaussian kernels (σ = 2.55 mm) using Connectome Workbench command line utilities (Glasser et al., 2013). Signals were normalized (z-scored). This yielded time courses representative of the entire cortical surface, subcortex (accumbens, amygdala, caudate, hippocampus, pallidum, putamen, and thalamus), and cerebellum, but excluding non-gray matter tissue. Signals from adjacent cortex were regressed from subcortical voxels, as in Datasets 1 and 2.

Analysis

Mapping Network Structure and Identifying Large-Scale Networks:

The network organization of each participant's brain was delineated following (44, 45) using the Infomap algorithm for community detection (46). In this approach, we calculated the pair-wise (zero-lag) cross-correlation matrix of the time courses from all brain vertices (on the cortical surfaces) and voxels (in subcortical structures), concatenated across sessions. Correlations between vertices/voxels within 30 mm of each other were set to zero in this matrix to avoid basing network membership on correlations attributable to

local spatial autocorrelation, which allows distributed brain networks to be identified using these approaches(47). Testing indicated that the exact value of this parameter within a range of ~10mm to 50mm does not affect identified brain networks. Geodesic distance was used for within-hemisphere surface connections (because smoothing was performed geodesically along the surface) and Euclidean distance for subcortical-to-cortical connections that could be affected by volumetric smoothing of subcortical voxels. Connections between subcortical structures were disallowed, as we observed extremely high correlation values within nearly the entire basal ganglia that would prevent network structures from emerging. Interhemispheric connections between the cortical surfaces were retained, as smoothing was not performed across the midsagittal plane.

We observed that connectivity patterns within regions known to have low BOLD signal due to susceptibility artifact dropout (e.g., ventral anterior temporal lobe and portions of orbitofrontal cortex) were unstructured and inconsistent across individuals. To avoid having the delineated network structures distorted by regions with poor signal, connections to regions with average mode-1000 normalized BOLD signal <750 were set to zero (as in (48, 49)).

The cross-correlation matrix was then thresholded to retain at least the strongest X% of connections to each vertex and voxel where we varied X from 0.01% to 5% (1, 44, 45). Note that this thresholding approach differs from previous approaches for forming brain graphs from functional connectivity data (e.g., (6, 47)). The typical procedure applies a uniform edge density threshold to all functional connectivity values in the brain. The weakness of the uniform threshold approach is that subcortical structures generally have decreased BOLD signal-to-noise relative to cortex due to their greater distance from the MR head coil. The result is that functional connectivity patterns seeded from subcortical voxels have weaker peak connectivity strengths, even though they may appear well organized and coherent with known cortical networks. Thus, with a uniform threshold, these regions are frequently not identified as being networked with cortical regions.

Each thresholded matrix formed using each of the separate density thresholds described above was used as the input for the Infomap algorithm, which employs many iterations of a random walker that traverses between connected cells of the matrix, and identifies communities based on the amount of time spent traversing within the same sets of nodes. Infomap calculated community assignments separately for each threshold. The primary community assignments of interest were those derived from the 0.1% density threshold, which we have shown in prior work well subdivides large-scale networks into more internally homogenous and externally valid subnetworks(45). Thus, the infomap-derived communities calculated using this threshold represent subnetworks in the brain. Small subnetworks with 10 or fewer vertices/voxels were considered unassigned and removed from further consideration. The above analysis was conducted in each individual participant.

Mapping Individual-Specific Large-Scale Networks

While communities derived from sparse thresholds represent subnetworks, communities derived from denser thresholds represent more traditional large-scale brain networks(45, 47). Thus, we identified the set of canonical large-scale networks in each participant's brain using the results of the infomap community detection algorithm described above, combining across all tested thresholds. At each threshold, the algorithm returned community identities for each vertex and voxel. Communities were labeled by matching them at each threshold to a set of independent group average networks, described in (6). Briefly, these networks were computed by applying the Infomap algorithm across multiple thresholds to data averaged vertexwise across an independent dataset of 120 healthy individuals (data available here: https://openneuro.org/datasets/ds000243/versions/00001). See (6) for details. These group averaged networks are now publicly available as part of the Network Correspondence Toolbox(50) (https://github.com/rubykong/cbig_network_correspondence_data/tree/master/atlases/fs_LR_32k/WashU). The matching approach proceeded as follows: 1) At each density threshold, all identified communities were compared with the independent group networks using the Jaccard Index of spatial overlap. 2) The community with the best match (highest overlap) to one of the independent networks was assigned that network identity, and then not considered for further comparison with other independent networks within that threshold. Matches lower than Jaccard = 0.1 were not considered (to avoid matching based on only a few vertices). Matches were first made with the large, well-known networks (in order: default, lateral visual, motor hand, motor mouth, frontoparietal, action mode, dorsal attention, language), and then to the smaller networks (salience, parietal memory, contextual association, medial visual, motor foot, and somato-cognitive action). In each individual and in the average, a "consensus" network assignment was derived by collapsing assignments across thresholds, giving each node the assignment it had at the sparsest possible threshold at which it was successfully assigned to one of the known group networks.

Task Analysis

Task evoked activations were modeled individually for each vertex and voxel with a general linear model (GLM) (51), using in-house image analysis software written in IDL (Research Systems, Inc.). First level GLM analyses were conducted separately for each session in a given participant, and second level within-participant analyses were run on the session-wise beta values of a single participant. Planned second-level contrasts were evaluated as paired voxel/vertex-wise t-tests comparing these beta values, and the resulting t-values in each voxel/vertex were then Z-transformed for further analysis.

We included tasks that had two different types of designs (motor = block design, spatial/verbal discrimination = mixed block/event-related design). In the block design motor task, a block regressor was convolved with a canonical hemodynamic response to model the five experimental conditions: tongue, left hand, right hand, left foot, right foot.

The spatial and verbal discrimination tasks were jointly modeled in a mixed block-event related design. Events were modeled with a finite impulse response model (as above, with 8 timepoints for each event); separate event regressors were included for the start and end cues in each task, and for the different trial types (noun, verb, 50% coherence, 0% coherence). The block (sustained activity) was modeled with a square block regressor of the duration of the block of events of that type (140 seconds), with separate regressors for sustained activity in the semantic and coherence task. Given the low number of error trials, errors were not modeled in any task. In addition to these terms, constant and linear effects were modeled for each run.

Calculating Subnetwork Time Delays

We computed lagged correlation estimates by extending a previously published method(52) to create a "lag map" for each AMN subnetwork in each subject representing the time delay of each vertex relative to the subnetwork. First, within each separate BOLD run, data was interpolated into motion-censored frames using frequency content from uncensored data(23), estimated based on the Lomb-Scargle periodogram(53). Run time course signals for all vertices in the cortex were then temporally upsampled to an interval of 0.03 seconds using a sinc interpolation, to enable more precise estimates of signal lead/lags. We then averaged BOLD signals across all voxels/vertices within each of the three subnetworks, and we computed a lagged cross-covariance function between each subnetwork time course and the time course of every other vertex in the cortex. Cross-covariance estimates were computed with a maximum lead/lag of 3.0 seconds. These cross-covariance estimates were then averaged across runs. For each vertex, we then computed the temporal offset that resulted in the strongest covariance with the subnetwork signal; this was taken as the signal lead/lag between the subnetwork and that vertex. This resulted in a lag map for each subnetwork. We note that varying the upsampling interval did not affect the general topology or values in these lag maps, except that larger intervals resulted in less precise lag estimates due to the limited precision of possible lag values (see Figure S7). In our experience, with these data quantities, lag correlation estimates between two signals are unstable when the zero-lag correlation between them is less than R = 0.1. Therefore, vertices with connectivity strengths to the subnetwork of less than R = 0.1 were excluded from all subsequent analyses. (Applying a range of values for this parameter— from -0.1 to 0.3—did not meaningfully impact results).

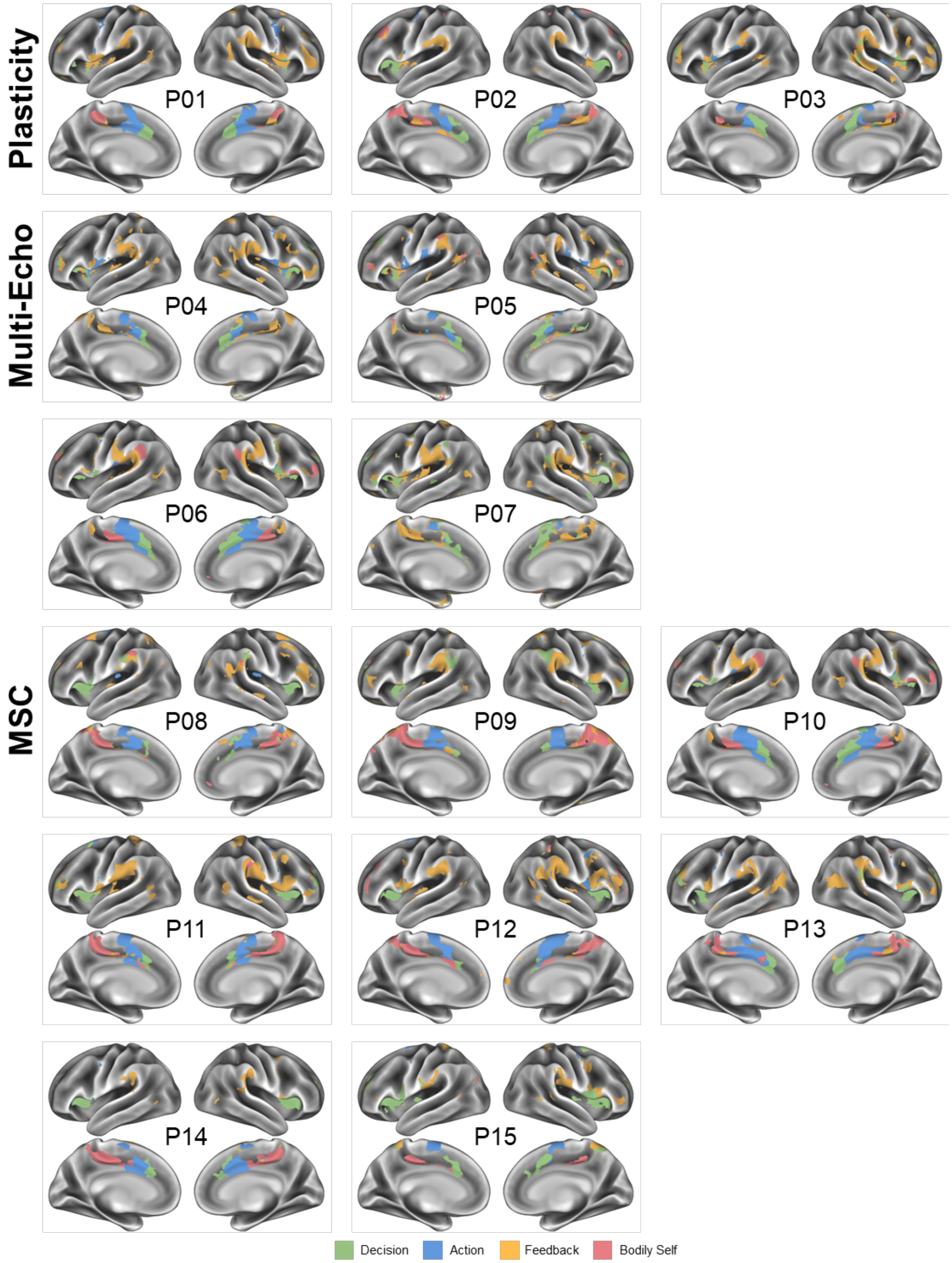

Figure S1: Individual-specific AMN subnetwork maps. Maps illustrate the topography of the four AMN subnetworks in each participant within the Plasticity dataset (N=3, 2F, ages 25-35 years), the Multi-Echo dataset (N=4, 0F, ages 24-39), and the Midnight Scan Club (MSC) dataset (N=8, 4F, ages 24-34).

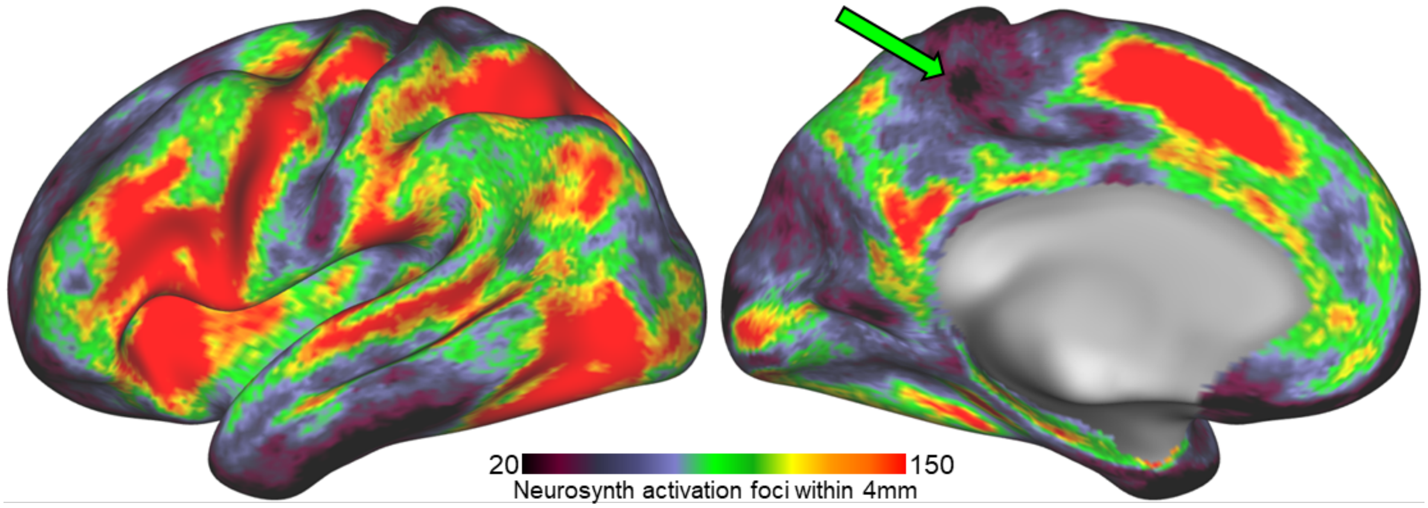

Figure S2: Regional coverage of Neurosynth activation foci. We projected all activation foci within the Neurosynth database to the cortical vertices of the Conte69 atlas surface within 4 mm. The map illustrates the number of activation foci mapped to each vertex. Specific regions of cortex exhibited few activation foci, indicating that few studies within the Neurosynth database activated that region of the brain. Aside from susceptibility artifact regions in the ventral temporal and orbitofrontal cortices, the pars marginalis of the cingulate sulcus (green arrow) exhibited some of the least frequent activation out of all cortical vertices.

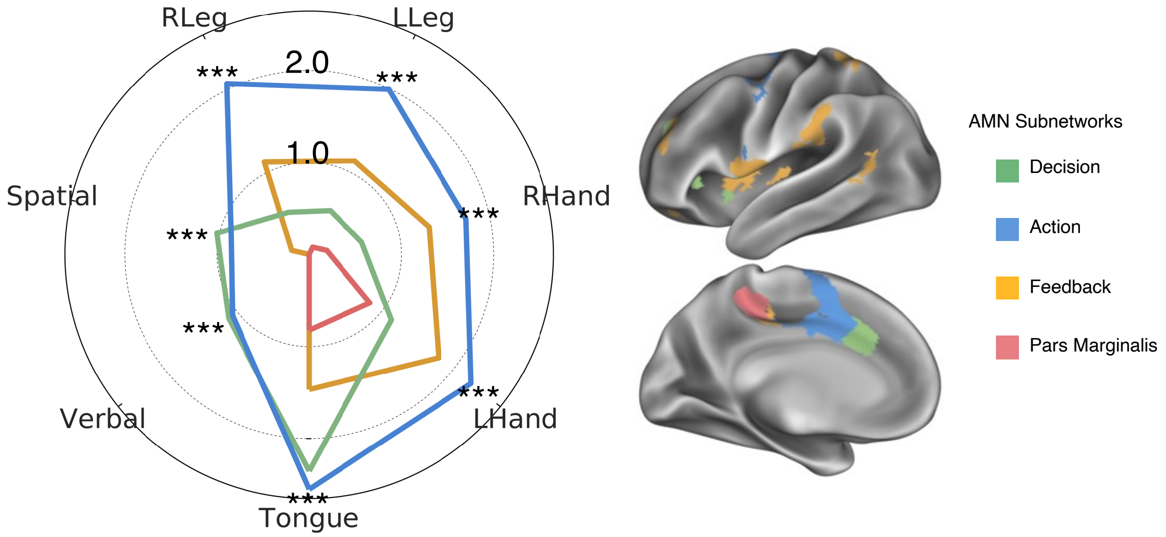

Figure S3: Differentiation of AMN subnetworks based on task activations. Ten participants performed motor tasks(11) (including flexure of right and left toes, open/closing of right and left hand, and left-right movement of the tongue), as well as a spatial and a verbal discrimination task(12). The radial axis indicates the z-score of each condition relative to baseline fixation, averaged across all participants and across all vertices in each individual-specific AMN subnetwork. Significant differences among subnetwork activations in each condition, determined via ANOVA, are indicated as *** - p(corr.) < 0.001. Inset shows subnetwork topography for the exemplar participant (P01).

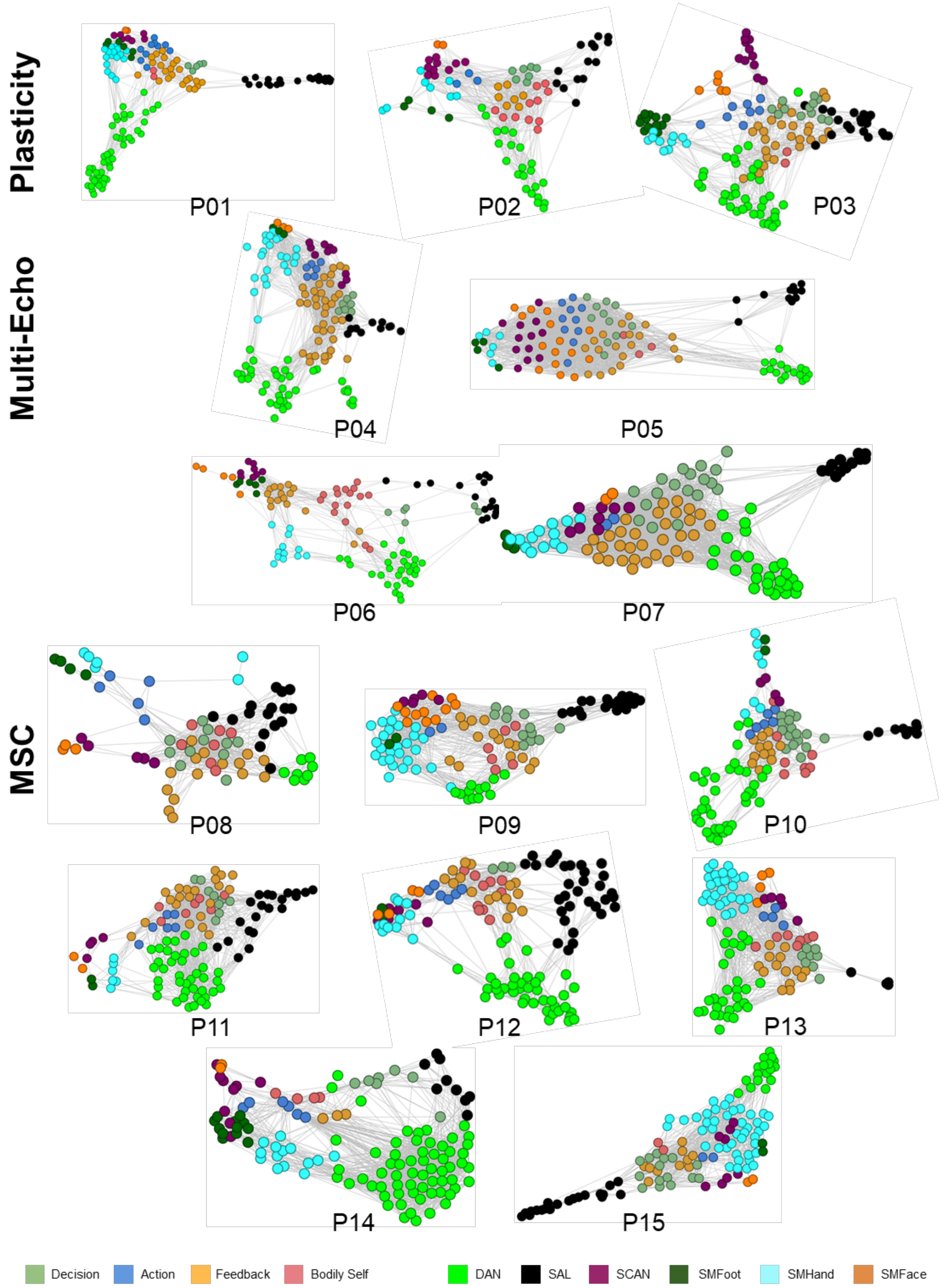

Figure S4: Individual AMN subnetwork spring embedding plots. Spring-embedding plots illustrate connections between the four AMN Subnetworks and the Salience (black), DAN (bright green), SCAN (red), Foot (dark green), Hand (cyan), and Mouth (orange) networks. Plots are shown for each subject in the Plasticity dataset (N=3, 2F, ages 25-35 years), the Multi-Echo dataset (N=4, 0F, ages 24-39), and the Midnight Scan Club (MSC) dataset (N=8, 4F, ages 24-34).

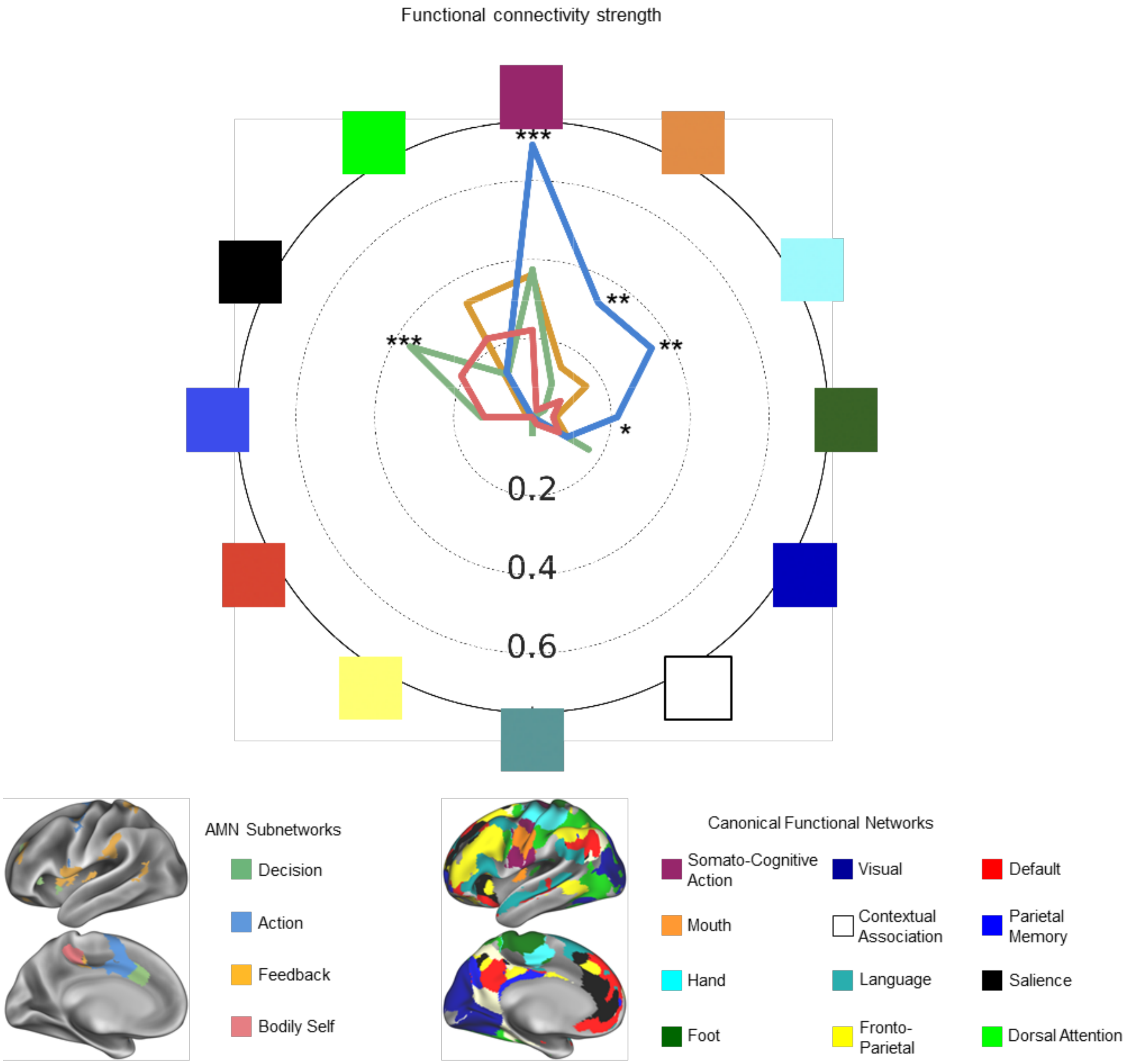

Figure S5: Functional connectivity patterns of AMN subnetworks, excluding local connections. To ensure that FC values were not driven by local autocorrelations of the fMRI signal, we computed subnetwork-to-network functional connectivity after excluding all functional connections between cortical vertices within 20 mm of each other. Across participants, individual-specific action-mode subnetworks still demonstrate preferential connectivity to other individual-specific functional networks, with the same pattern observed in Fig 4. The radial axis indicates the strength of functional connectivity Z(r) between each AMN subnetwork and each canonical functional network. Negative connectivity values are not represented. Significant differences among subnetwork connectivities to each network, determined via ANOVA, are indicated as *** - p(corr.) < 0.001; ** - p(corr.) < 0.01; * - p(corr.) < 0.05. Colors and spatial locations of AMN subnetworks (left) and other canonical networks (right) are shown in the exemplar participant at the bottom.

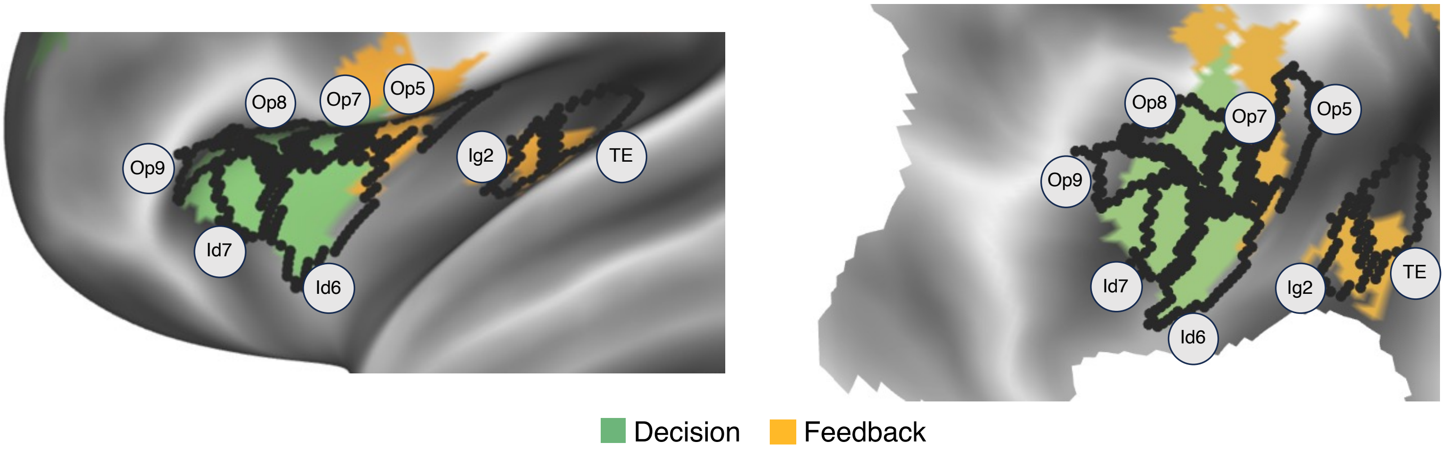

Figure S6: AMN subnetworks in dysgranular and granular insula. Overlap of Decision (green) and Feedback (yellow) group average subnetwork locations with insular and opercular architectonic areas (black borders). Overlap is shown on an inflated cortical surface (left) as well as on a flat map (right). The decision subnetwork (green) was restricted to dysgranular insula areas (Id7 and Id8), while the Feedback area overlapped with a granular area (Ig2). Op – Opercular areas; Id – dysgranular Insula areas; Ig – granular Insula areas; TE – area Te 1.0.

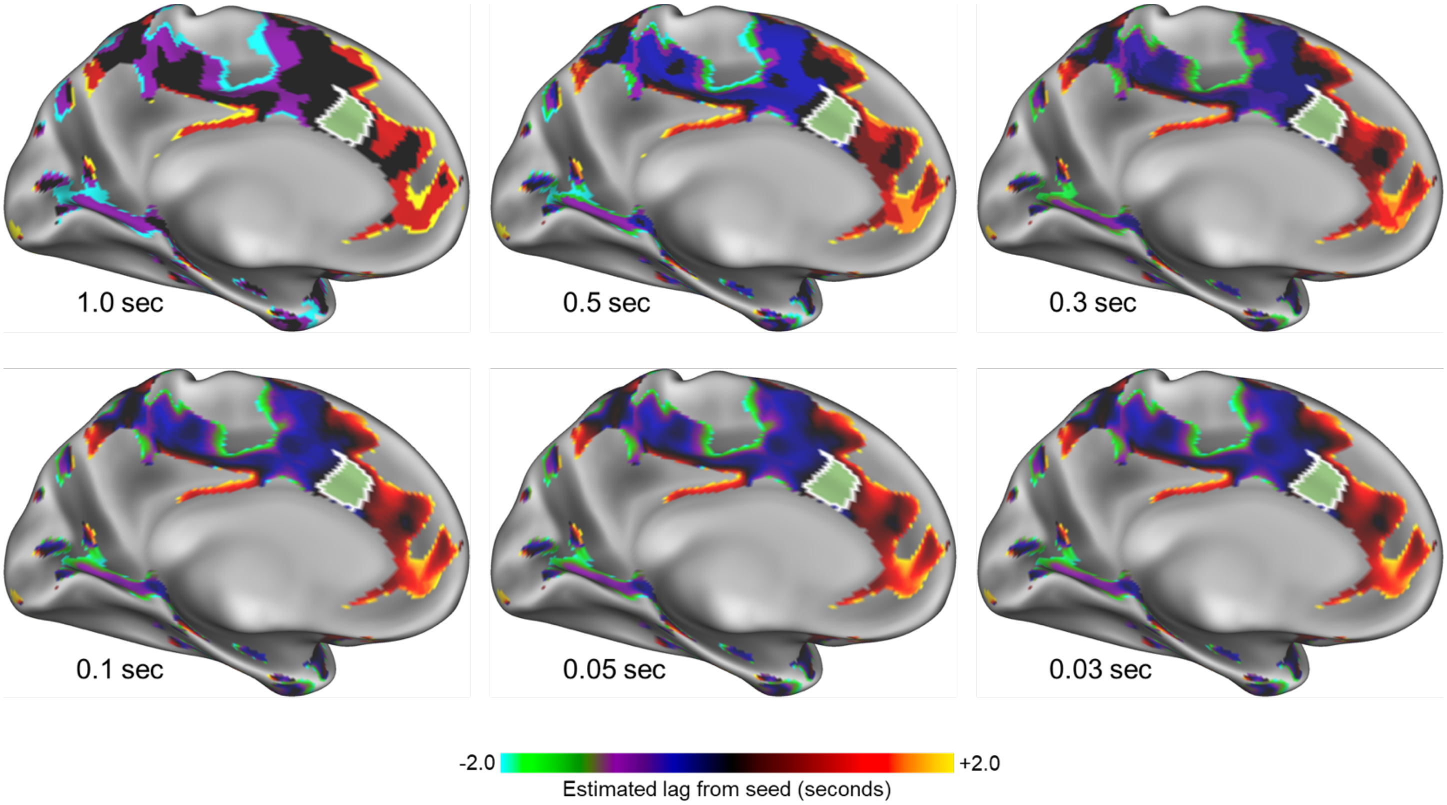

Figure S7: Maps of temporal lag are similar across levels of temporal upsampling. Estimated temporal lag for each vertex from the Anterior (Decision) AMN subnetwork in an example subject (green with white outline). Each map represents lags estimated from fMRI (BOLD data) that has been temporally upsampled to various levels from 1 second (top left) to 0.03 seconds (bottom right). Maps were very similar across upsampling levels, but increased upsampling allowed observation of finer spatial detail in the lag map.

Table S1: Average locations of AMN subnetworks. MNI coordinates of peak cross-subject overlap in various structures within each AMN subnetwork.

Structure	Left	Right
**Decision (Anterior)**		
Dorsal anterior cingulate	-3 21 28	10 17 33
Anterior insula	-33 11 12	34 16 11
Anterior prefrontal cortex	-28 42 24	29 43 21
**Action (Central)**		
Dorsal anterior cingulate	-10 2 40	10 3 46
Supplementary motor area	-10 -8 64	10 -6 66
**Feedback (Lateral)**		
Supramarginal gyrus	-60 -35 35	56 -27 31
Anterior/middle insula	-34 0 14	37 0 12
Inferior frontal sulcus	-36 39 10	47 39 2
Inferior frontal gyrus	-50 7 8	55 8 7
**Bodily Self (Pars Marginals)**		
Pars marginalis of cingulate	-16 -33 42	15 -36 49

Table S2: AMN Subnetwork vs Large Scale Network Connectivity. Paired t-tests compared functional connectivity to a large-scale network between two different AMN subnetworks. Asterisks indicate significant differences (FDR corrected to alpha < 0.05) between pairs of subnetworks.

	Subnetwork comparison					
Connectivity to:	Feedback v Decision	Feedback v Action	Feedback v Bodily Self	Decision v Action	Decision v Bodily Self	Action v Bodily Self
Default Mode	T(14)=0.46; p=0.65	T(14)=-3.66; p=0.003*	T(12)=-3.11; p=0.009*	T(14)=-3.22; p=0.006*	T(12)=-2.97; p=0.012*	T(12)=1.22; p=0.24
SCAN	T(14)=-0.31; p=0.76	T(14)=-6.81; p<0.001*	T(12)=1.59; p=0.13	T(14)=-5.01; p<0.001*	T(12)=2.23; p=0.05	T(12)=5.32; p<0.001*
Visual	T(14)=-1.80; p=0.09	T(14)=0.61; p=0.55	T(12)=-0.11; p=0.91	T(14)=1.75; p=0.10	T(12)=1.37; p=0.20	T(12)=-0.34; p=0.74
Fronto-Parietal	T(14)=-0.26; p=0.8	T(14)=0.71; p=0.49	T(12)=0.60; p=0.56	T(14)=0.86; p=0.40	T(12)=0.86; p=0.41	T(12)=-0.31; p=0.76
Dorsal Attention	T(14)=4.30; p=0.001*	T(14)=5.07; p<0.001*	T(12)=2.07; p=0.06	T(14)=0.16; p=0.88	T(12)=-3.71; p=0.003*	T(12)=-2.33; p=0.04
Language	T(14)=-2.38; p=0.03	T(14)=-2.13; p=0.05	T(12)=4.42; p=0.001*	T(14)=0.96; p=0.35	T(12)=6.17; p<0.001*	T(12)=6.40; p<0.001*
Salience	T(14)=-7.39; p<0.001*	T(14)=2.02; p=0.06	T(12)=-3.87; p=0.002*	T(14)=7.61; p<0.001*	T(12)=1.61; p=0.13	T(12)=-4.62; p=0.001*
Somatomotor Foot	T(13)=3.81; p=0.002*	T(13)=-5.53; p<0.001*	T(11)=-0.01; p=0.99	T(13)=-6.98; p<0.001*	T(11)=-1.88; p=0.09	T(11)=4.48; p=0.001*
Somatomotor Hand	T(14)=3.42; p=0.004*	T(14)=-4.96; p<0.001*	T(12)=1.18; p=0.26	T(14)=-6.64; p<0.001*	T(12)=-1.14; p=0.28	T(12)=7.28; p<0.001*
Somatomotor Mouth	T(14)=1.39; p=0.19	T(14)=-4.51; p<0.001*	T(12)=2.40; p=0.03	T(14)=-7.43; p<0.001*	T(12)=1.21; p=0.25	T(12)=4.68; p=0.001*
Parietal Memory	T(14)=-5.44; p<0.001*	T(14)=1.27; p=0.22	T(12)=-5.95; p<0.001*	T(14)=5.71; p<0.001*	T(12)=0.70; p=0.50	T(12)=-6.79; p<0.001*
Context	T(13)=0.02; p=0.98	T(13)=0.05; p=0.97	T(11)=-5.53; p<0.001*	T(13)=0.02; p=0.99	T(11)=-5.94; p<0.001*	T(11)=-3.84; p=0.003*

Table S3: Temporal ordering of signals between AMN subnetworks and other networks. T-tests describing differences in temporal ordering between pairs of networks and subnetworks.

	Salience	Decision	Bodily Self	Feedback	Action	SCAN
Decision	t(14) = -3.97; p = 0.001					
Pars Marg.	t(14) = -3.29; p = 0.006	t(14) = 0.36; p = 0.72				
Feedback	t(14) = -4.55; p < 0.001	t(14) = -0.26; p = 0.80	t(14) = -1.05; p = 0.31			
Action	t(14) = -5.69; p < 0.001	t(14) = -4.12; p = 0.001	t(14) = -4.08; p = 0.002	t(14) = -4.28; p = 0.001		
SCAN	t(14) = -4.19; p = 0.001	t(14) = -2.53; p = 0.02	t(14) = -2.63; p = 0.02	t(14) = -2.36; p = 0.03	t(14) = 0.22; p = 0.83	
Somatomotor Hand	t(14) = -3.82; p = 0.002	t(14) = -2.38; p = 0.03	t(14) = -2.57; p = 0.02	t(14) = -2.22; p = 0.04	t(14) = -0.78; p = 0.45	t(14) = -1.02; p = 0.32

Table S4: Task Activation differences between AMN subnetworks. Paired t-tests compared activation vs baseline in each task between two different AMN subnetworks. Asterisks indicate significant differences in activation between two subnetworks (FDR-corrected to alpha < 0.05) for a specific task condition.

	Subnetwork comparison					
Task:	Feedback vs Decision	Feedback vs Action	Feedback vs Bodily Self	Decision vs Action	Decision vs Bodily Self	Action vs Bodily Self
Tongue	T(9)=-4.57; p=0.001*	T(9)=-6.68; p<0.001*	T(9)=2.45; p=0.04*	T(9)=-0.84; p=0.42	T(9)=6.81; p<0.001*	T(9)=9.70; p<0.001*
Left Hand	T(9)=3.14; p=0.01*	T(9)=-2.59; p=0.03*	T(9)=6.71; p<0.001*	T(9)=-6.72; p<0.001*	T(9)=1.32; p=0.22	T(9)=7.54; p<0.001*
Right Hand	T(9)=2.88; p=0.02*	T(9)=-1.91; p=0.09	T(9)=7.11; p<0.001*	T(9)=-4.93; p=0.001*	T(9)=1.06; p=0.32	T(9)=5.59; p<0.001*
Left Foot	T(9)=2.25; p=0.05	T(9)=-2.74; p=0.02*	T(9)=5.11; p=0.001*	T(9)=-5.04; p=0.001*	T(9)=1.69; p=0.13	T(9)=5.11; p=0.001*
Right Foot	T(9)=2.45; p=0.04*	T(9)=-3.12; p=0.01*	T(9)=6.44; p<0.001*	T(9)=-4.54; p=0.001*	T(9)=2.47; p<0.04	T(9)=6.79; p<0.001*
Spatial Discrimination	T(9)=-4.31; p=0.002*	T(9)=-4.56; p=0.001*	T(9)=2.45; p=0.04*	T(9)=0.96; p=0.36	T(9)=5.80; p<0.001*	T(9)=6.00; p<0.001*
Verbal Discrimination	T(9)=-5.54; p<0.001*	T(9)=-4.90; p=0.001*	T(9)=6.54; p<0.001*	T(9)=0.51; p=0.63	T(9)=9.83; p<0.001*	T(9)=8.56; p<0.001*

Supplemental References:

1. E. M. Gordon, *et al.*, A somato-cognitive action network alternates with effector regions in motor cortex. *Nature* **617**, 351–359 (2023).
2. D. J. Newbold, *et al.*, Plasticity and Spontaneous Activity Pulses in Disused Human Brain Circuits. *Neuron* **107**, 580–89 (2020).
3. D. J. Newbold, *et al.*, Cingulo-opercular control network and disused motor circuits joined in standby mode. *Proc. Natl. Acad. Sci.* **118**, e2019128118 (2021).
4. N. A. Seider, *et al.*, Accuracy and Reliability of Diffusion Imaging Models. *NeuroImage* **254**, 119138 (2022).
5. N. U. F. Dosenbach, *et al.*, Real-time motion analytics during brain MRI improve data quality and reduce costs. *NeuroImage* **161**, 80–93 (2017).
6. E. M. Gordon, *et al.*, Precision Functional Mapping of Individual Human Brains. *Neuron* **95**, 791–807 (2017).
7. C. Gratton, *et al.*, Functional brain networks are dominated by stable group and individual factors, not cognitive or daily variation. *Neuron* **98**, 439–452 (2018).
8. S. Marek, *et al.*, Spatial and Temporal Organization of the Individual Human Cerebellum. *Neuron* **100**, 977–993 (2018).
9. C. M. Sylvester, *et al.*, Individual-specific functional connectivity of the amygdala: A substrate for precision psychiatry. *Proc. Natl. Acad. Sci.* **117**, 3808–18 (2020).
10. D. J. Greene, *et al.*, Integrative and Network-Specific Connectivity of the Basal Ganglia and Thalamus Defined in Individuals. *Neuron* **105**, 742–758 (2020).
11. D. M. Barch, *et al.*, Function in the human connectome: Task-fMRI and individual differences in behavior. *NeuroImage* **80**, 169–189 (2013).
12. J. W. Dubis, J. S. Siegel, M. Neta, K. M. Visscher, S. E. Petersen, Tasks Driven by Perceptual Information Do Not Recruit Sustained BOLD Activity in Cingulo-Opercular Regions. *Cereb. Cortex* **26**, 192–201 (2016).
13. L. Glass, Moiré effect from random dots. *Nature* **223**, 578–580 (1969).
14. Y. Zhang, M. Brady, S. Smith, Segmentation of brain MR images through a hidden Markov random field model and the expectation-maximization algorithm. *IEEE Trans. Med. Imaging* **20**, 45–57 (2001).
15. J. Talairach, P. Tournoux, *Co-planar stereotaxic atlas of the human brain* (Thieme Medical Publishers, Inc, 1988).
16. J. L. Lancaster, *et al.*, A modality-independent approach to spatial normalization of tomographic images of the human brain. *Hum. Brain Mapp.* **3**, 209–223 (1995).
17. A. M. Dale, B. Fischl, M. I. Sereno, Cortical Surface-Based Analysis: I. Segmentation and Surface Reconstruction. *NeuroImage* **9**, 179–194 (1999).
18. B. Fischl, M. I. Sereno, A. M. Dale, Cortical Surface-Based Analysis: II: Inflation, Flattening, and a Surface-Based Coordinate System. *NeuroImage* **9**, 195–207 (1999).
19. D. C. Van Essen, M. F. Glasser, D. L. Dierker, J. Harwell, T. Coalson, Parcellations and Hemispheric Asymmetries of Human Cerebral Cortex Analyzed on Surface-Based Atlases. *Cereb. Cortex* **22**, 2241–2262 (2012).
20. E. C. Robinson, *et al.*, MSM: a new flexible framework for Multimodal Surface Matching. *NeuroImage* **100**, 414–426 (2014).
21. T. O. Laumann, *et al.*, Functional System and Areal Organization of a Highly Sampled Individual Human Brain. *Neuron* **87**, 657–670 (2015).
22. R. Ciric, *et al.*, Benchmarking of participant-level confound regression strategies for the control of motion artifact in studies of functional connectivity. *NeuroImage* **154**, 174–187 (2017).
23. J. D. Power, *et al.*, Methods to detect, characterize, and remove motion artifact in resting state fMRI. *NeuroImage* **84**, 320–341 (2014).
24. C. Gratton, *et al.*, Removal of high frequency contamination from motion estimates in single-band fMRI saves data without biasing functional connectivity. *NeuroImage* **217**, 116866 (2020).
25. M. N. Hallquist, K. Hwang, B. Luna, The nuisance of nuisance regression: Spectral misspecification in a common approach to resting-state fMRI preprocessing reintroduces noise and obscures functional connectivity. *NeuroImage* **82**, 208–225 (2013).
26. J. D. Power, B. L. Schlaggar, S. E. Petersen, Recent progress and outstanding issues in motion correction in resting state fMRI. *NeuroImage* **105**, 536–551 (2015).
27. B. Fischl, FreeSurfer. *NeuroImage* **62**, 774–781 (2012).

28. R. Patriat, E. K. Molloy, R. M. Birn, Using Edge Voxel Information to Improve Motion Regression for rs-fMRI Connectivity Studies. *Brain Connect.* **5**, 582–595 (2015).
29. T. D. Satterthwaite, *et al.*, An improved framework for confound regression and filtering for control of motion artifact in the preprocessing of resting-state functional connectivity data. *NeuroImage* **64**, 240–256 (2013).
30. Y. Behzadi, K. Restom, J. Liau, T. T. Liu, A component based noise correction method (CompCor) for BOLD and perfusion based fMRI. *NeuroImage* **37**, 90–101 (2007).
31. D. Marcus, *et al.*, Informatics and Data Mining Tools and Strategies for the Human Connectome Project. *Front. Neuroinformatics* **5**, 4 (2011).
32. M. F. Glasser, *et al.*, The minimal preprocessing pipelines for the Human Connectome Project. *NeuroImage* **80**, 105–124 (2013).
33. R. L. Buckner, F. M. Krienen, A. Castellanos, J. C. Diaz, B. T. T. Yeo, The organization of the human cerebellum estimated by intrinsic functional connectivity. *J. Neurophysiol.* **106**, 2322–2345 (2011).
34. D. J. Greene, *et al.*, Developmental Changes in the Organization of Functional Connections between the Basal Ganglia and Cerebral Cortex. *J. Neurosci.* **34**, 5842–5854 (2014).
35. C. J. Lynch, *et al.*, Rapid Precision Functional Mapping of Individuals Using Multi-Echo fMRI. *Cell Rep.* **33**, 108540 (2020).
36. J. D. Power, *et al.*, Distinctions among real and apparent respiratory motions in human fMRI data. *NeuroImage* **201**, 116041 (2019).
37. M. F. Glasser, D. C. Van Essen, Mapping Human Cortical Areas In Vivo Based on Myelin Content as Revealed by T1- and T2-Weighted MRI. *J. Neurosci.* **31**, 11597–11616 (2011).
38. D. N. Greve, B. Fischl, Accurate and robust brain image alignment using boundary-based registration. *NeuroImage* **48**, 63–72 (2009).
39. M. Jenkinson, C. F. Beckmann, T. E. J. Behrens, M. W. Woolrich, S. M. Smith, FSL. *NeuroImage* **62**, 782–790 (2012).
40. P. Kundu, S. J. Inati, J. W. Evans, W.-M. Luh, P. A. Bandettini, Differentiating BOLD and non-BOLD signals in fMRI time series using multi-echo EPI. *NeuroImage* **60**, 1759–1770 (2012).
41. P. Kundu, *et al.*, Integrated strategy for improving functional connectivity mapping using multiecho fMRI. *Proc. Natl. Acad. Sci.* **110**, 16187–16192 (2013).
42. S. Posse, *et al.*, Enhancement of BOLD-contrast sensitivity by single-shot multi-echo functional MR imaging. *Magn. Reson. Med.* **42**, 87–97 (1999).
43. J. D. Power, K. A. Barnes, A. Z. Snyder, B. L. Schlaggar, S. E. Petersen, Spurious but systematic correlations in functional connectivity MRI networks arise from subject motion. *NeuroImage* **59**, 2142–54 (2012).
44. E. M. Gordon, *et al.*, Individualized Functional Subnetworks Connect Human Striatum and Frontal Cortex. *Cereb. Cortex* **32**, 2868–2884 (2022).
45. E. M. Gordon, *et al.*, Default-mode network streams for coupling to language and control systems. *Proc. Natl. Acad. Sci.* **117**, 17308–17319 (2020).
46. M. Rosvall, C. T. Bergstrom, Maps of random walks on complex networks reveal community structure. *Proc. Natl. Acad. Sci.* **105**, 1118–1123 (2008).
47. J. D. Power, *et al.*, Functional Network Organization of the Human Brain. *Neuron* **72**, 665–678 (2011).
48. E. M. Gordon, *et al.*, Generation and Evaluation of a Cortical Area Parcellation from Resting-State Correlations. *Cereb. Cortex* **26**, 288–303 (2016).
49. G. S. Wig, T. O. Laumann, S. E. Petersen, An approach for parcellating human cortical areas using resting-state correlations. *NeuroImage* **93**, 276–291 (2014).
50. R. Kong, *et al.*, A network correspondence toolbox for quantitative evaluation of novel neuroimaging results. *Nat. Commun.* **16**, 2930 (2025).
51. F. M. Miezin, L. Maccotta, J. M. Ollinger, S. E. Petersen, R. L. Buckner, Characterizing the Hemodynamic Response: Effects of Presentation Rate, Sampling Procedure, and the Possibility of Ordering Brain Activity Based on Relative Timing. *NeuroImage* **11**, 735–759 (2000).
52. A. Mitra, A. Z. Snyder, C. D. Hacker, M. E. Raichle, Lag structure in resting-state fMRI. *J. Neurophysiol.* **111**, 2374–2391 (2014).
53. N. R. Lomb, Least-squares frequency analysis of unequally spaced data. *Astrophys. Space Sci.* **39**, 447–462 (1976).
